# Anti-inflammatory effect of a *Bacillus halotolerans* DY299 strain isolated from Arshan on methicillin-resistant *Staphylococcus aureus*-induced atopic dermatitis in mouse

**DOI:** 10.3389/fmicb.2026.1929286

**Published:** 2026-09-22

**Authors:** Caixia Deng, Yan Zhao, Qishi Qishi, Lei Wang, Lili Bao, Yingsong Chen, Dezhi Yang

**Affiliations:** 1 Engineering of the Ministry of Education of Mongolian Medicine, Inner Mongolia Minzu University, Tongliao, China; 2 National and Local Joint Engineering Research Center of Modern Mongolian Medicine Research and Testing, International Mongolian Hospital of Inner Mongolia, Hohhot, China; 3 Hohhot Stomatological Hospital (Inner Mongolia Autonomous Region Stomatological Hospital), Hohhot, Inner Mongolia, China; 4 School of Basic Medical Sciences, Inner Mongolia Medical University, Hohhot, China

**Keywords:** atopic dermatitis, *Bacillus halotolerans*, biofilm, DY299-CFS, MRSA, therapeutic effect

## Abstract

**Introduction:**

Methicillin-resistant *Staphylococcus aureus* (MRSA) colonization is a major contributor to atopic dermatitis (AD), and its rising resistance and virulence underscore the urgent need for novel antimicrobial strategies.

**Methods:**

In this study, a halotolerant strain designated *Bacillus halotolerans* DY299 was isolated from Arshan water. The cell-free culture supernatant (DY299-CFS) was prepared and its anti-biofilm and antibacterial activities against MRSA were evaluated *in vitro*. The therapeutic efficacy was assessed in an MRSA-induced AD mouse model, with skin lesion severity, stratum corneum hydration, mast cell infiltration, and cutaneous MRSA colonization examined. Serum levels of IgE, IL-13, IFN-γ, and IL-17 were measured by ELISA. To elucidate the molecular mechanisms, whole-genome sequencing of DY299, LC-MS/MS metabolomic profiling of its supernatant, transcriptomic analysis of skin tissues, and network pharmacology-based target prediction were integrated.

**Results:**

DY299-CFS demonstrated significant anti-biofilm activity, achieving an inhibition rate of 50.93% and effectively eradicating MRSA biofilm both *in vitro* and *in vivo*. Topical DY299-CFS treatment markedly alleviated inflammatory skin lesions, improved stratum corneum hydration, reduced mast cell infiltration, and suppressed cutaneous MRSA colonization in AD mice. ELISA assays revealed that DY299-CFS significantly decreased serum levels of IgE, IL-13, IFN-γ, and IL-17 compared with the model group, indicating concurrent suppression of Th2-type and Th1/Th17-type immune responses.

**Discussion:**

Multi-omics analysis collectively identified the IL-17 signaling pathway as a key mediator through which DY299-CFS exerts its anti-inflammatory effects against MRSA-induced skin inflammation. Collectively, these findings establish *Bacillus halotolerans* DY299 as a promising anti-biofilm agent against MRSA and a valuable microbial resource for developing novel therapeutics for biofilm-driven inflammatory skin diseases such as AD, offering a multifaceted strategy that combines direct antimicrobial action with immunomodulatory effects.

## 1 Introduction

Atopic dermatitis (AD) is a chronic inflammatory dermatological disorder that is frequently complicated by the colonization of the skin by *Staphylococcus aureus* (*S. aureus*) (Sivori et al., 2024; Weidinger et al., 2018). In patients with AD, *S. aureus* colonizes the skin surface and forms biofilms, which have been shown to correlate with disease severity, treatment efficacy, and relapse frequency (Sivori et al., 2024). Methicillin-resistant *Staphylococcus aureus* (MRSA) is an important pathogen that is often implicated in recurrent infections of the skin and soft tissues in individuals with AD (Turner et al., 2019). The prevalence of biofilm-forming *S. aureus* in AD lesions has been demonstrated to enhance antibiotic resistance, thereby exacerbating the chronicity of the disease and rendering it more refractory to conventional therapeutic interventions (Jomehzadeh and Emrani, 2025).

In recent years, the use of probiotics and their metabolites for the management of skin diseases has become a focus of intensive research. Oral or topical administration of certain strains of Lactobacillus, Bifidobacterium, and *Bacillus* can alleviate the symptoms of atopic dermatitis through multiple mechanisms, including modulation of the microbiota, competitive exclusion, secretion of antimicrobial substances, and immune regulation (Holowacz et al., 2018; Kim et al., 2018). However, the efficacy of different strains varies considerably due to differences in species and strain characteristics. Therefore, targeted screening of highly potent functional strains from diverse habitats represents a key strategy for identifying ideal candidates. The genus *Bacillus* is widely distributed in soil, water, and extreme environments, and has drawn considerable attention owing to its abundant and stable metabolites. Previous studies have shown that cell-free supernatant of *Bacillus velezensis* not only exerts potent inhibitory effects against MRSA, but also yields heat-stable, acid- and alkali-tolerant antimicrobial peptides that kill bacteria by disrupting cell membranes. *In vivo* experiments have further demonstrated a significant reduction in infection-related mortality, underscoring the potential of this strain as an anti-infective probiotic (Baharudin et al., 2021).

*Bacillus* spp. are a group of Gram-positive, non-pathogenic bacteria that have garnered significant scientific interest as potential sources of novel natural products (Elshaghabee et al., 2017). This interest is primarily attributed to their remarkable ability to survive and thrive in high-salt environments. The capacity of *Bacillus* spp. to maintain their cellular structure and function under such extreme conditions has led to the evolution of salt-tolerant microorganisms that exhibit unique metabolic pathways, distinctive community characteristics, and the production of novel, specific metabolites (Golnari et al., 2024). These attributes collectively highlight the potential of *Bacillus* spp. to serve as valuable reservoirs for the discovery of bioactive compounds with potential applications in various fields, including pharmaceuticals and biotechnology (Mu and Cong, 2019). However, the antibacterial activity of *Bacillus* against MRSA and its ameliorative effects on inflammatory and immune indicators associated with AD remain unclear, and the underlying mechanisms have yet to be systematically elucidated.

Although previous studies have demonstrated the anti-MRSA potential of certain *Bacillus* strains, several knowledge gaps remain regarding the anti-biofilm activity of halotolerant Bacillus species from extreme environments, their therapeutic efficacy in MRSA-associated atopic dermatitis, and the underlying molecular mechanisms. To address these gaps, we isolated a halotolerant strain, *Bacillus halotolerans* DY299, from Arshan water in Hulunbuir, China. The novelty of this study lies in the systematic evaluation of its anti-biofilm and antibacterial properties against MRSA, the first demonstration of its therapeutic efficacy in an MRSA-induced AD model with comprehensive immune profiling, and the elucidation of its mechanism through integrated transcriptomic and network pharmacology analyses targeting the IL-17 signaling pathways.

## 2 Materials and methods

### 2.1 Bacterial strains, medium, and growth conditions

The Methicillin-Resistant *Staphylococcus aureus* strain (USA300) used in this study was purchased from BeNa Culture Collection (BNCC361194). *S. aureus* cells were grown at 37 °C, Luria Bertani (LB) broth or LB-agar (Beef Extract 3 g/L, NaCl 5 g/L, Peptone 10 g/L, Agar 20 g/L). *Bacillus* cells were grown at 37 °C, Rich Medium (RM) broth or RM-agar (NaCl 50 g/L, MgSO_4_ 9.7 g/L, Na_3_C_6_H_5_O_7_ 3.0 g/L, KCl 2.0 g/L, CaCl_2_ 0.2 g/L, Peptone 10 g/L, Agar 15.0 g/L, Yeast extract 2.0 g/L), all reagents were purchased from Beijing Solarbio Science & Technology Co., Ltd., Beijing, China.

### 2.2 Bacteria strains isolation and characterization

The Bacteria strain was isolated from Arshan (117°40′6″E, 48°54′7″N) water samples by streaking in RM-medium, incubated at constant temperature at 37 °C for 12 h (Deng et al., 2024). Single colonies were picked based on colony morphology (shape, size, color), streaked repeatedly, and purified and incubated for three times to finally obtain pure cultures, and the strains were frozen in the refrigerator at −80 °C (skin milk).

### 2.3 16S rRNA gene-based identification and whole genome sequencing of bacteria

*Bacillus halotolerans* DY299 was collected by centrifugation at 12000 rpm/min for 2 min after incubation in RM-broth medium at 37 °C with shaking. The genomic DNA was extracted and used as a template for PCR amplification of the 16S rRNA gene using universal primers (626&627) (Song et al., 2020) for bacteria. The amplification system was as follows: 1 μL of DNA template, 10 μL of 2 × EasyTaq PCR SuperMix (AS111, Transgen, Beijing, China), 2 μL of primers, and 7 μL of ddH_2_O. The amplification conditions were as follows: 94 °C for 2 min, 94 °C for 30 s, 55 °C for 30 s, 72 °C for 2 min, 72 °C for 10 min, 35 cycles. The PCR reaction products were purified and sent to Shanghai Sangon Biotech Co., Ltd. for sequencing analysis. The sequencing results were subjected to a BLAST search on NCBI to strains with similar affinities. The 16S rDNA sequences of highly similar related strains were obtained, and the phylogenetic tree was constructed using MEGA (V.11) software.

Whole genome sequencing was sent to Beijing Novogene Co., Ltd., genomic DNA was extracted by STE method and sequenced using Nanopore PromethION platform and Illumina NovaSeq PE150 platform.

### 2.4 Preparation of cell-free culture supernatant

Inhibitory activity was conducted using a cell-free culture supernatant (CFS) of the isolated strain (pH, 7.0). *Bacillus halotolerans* DY299 was cultured in RM broth medium for 24 h, 48 h and 72 h. The supernatant was collected and centrifuged at 12000 rpm/min for 2 min to obtain the DY299-CFS, filtered through a 0.22 μm membrane to obtain 24-CFS (from 24 h), 48-CFS (from 48 h), and 72-CFS (from 72 h). 50 mL of each CFS were lyophilized by vacuum freeze drying, and the dried powder was redissolved in sterile water to a final volume of 5 mL and storage at 4 °C. Heat-inactivation of cell-free supernatant (CFS): To evaluate whether the antibiofilm activity of the CFS was associated with heat-sensitive bioactive compounds, heat-inactivated CFS was prepared. Briefly, the CFS was incubated in a water bath at 95 °C for 20 min.

### 2.5 Cell-free supernatants UHPLC–MS/MS analysis

UHPLC–MS/MS assayed compounds of the 24-CFS group, and RM medium was used as a control. UHPLC–MS/MS analyses were performed using a Vanquish UHPLC system (ThermoFisher, Germany) coupled with an Orbitrap Q ExactiveTM HF mass spectrometer or Orbitrap Q ExactiveTMHF-X mass spectrometer (Thermo Fisher, Germany) in Novogene Co., Ltd. (Beijing, China). Samples were injected onto a Hypersil Goldcolumn (100 × 2.1 mm, 1.9 μm) using a 12 min linear gradient at a flow rate of 0.2 mL/min. The eluents for the positive and negative polarity modes were eluent A (0.1% FA in Water) and eluent B (Methanol). The solvent gradient was set as follows: 2% B, 1.5 min; 2–85% B, 3 min; 85–100% B, 10 min; 100–2% B, 10.1 min; 2% B, 12 min. Q ExactiveTM HF mass spectrometer was operated in positive/negative polarity mode with spray voltage of 3.5 kV, capillary temperature of 320 °C, sheath gas flow rate of 35 psi and aux gas flow rate of 10 L/min, S-lens RF level of 60, Aux gas heater temperature of 350 °C.

### 2.6 Determining the effects of DY299-CFS on MRSA biofilm formation

The anti-biofilm activity of DY299 cell-free supernatant (CFS) against MRSA was evaluated using a crystal violet semi-quantitative adhesion method in a 96-well plate format, where 100 μL of MRSA cell suspension (1 × 10^6^ CFU/mL) was added to each well, and then different concentrations of DY299-CFS (corresponding to final volumes of 50, 25, 12.5, 6.25, 3.125, and 1.5625 μL per well) were added to the experimental groups, while the heat-inactivation control group received the same volumes of heat-treated DY299-CFS at the corresponding concentration gradient, and the medium control group received the same volumes of sterile RM broth at the corresponding concentration gradient instead of DY299-CFS, and gentamicin (G8170, Solarbio, Beijing, China) at a final concentration of 50 μg/mL was used as a positive control, with all wells then replenished to a final volume of 200 μL with LB medium and incubated at 37 °C for 24 h; after incubation, the culture medium was aspirated, and each well was washed three times with 200 μL of sterile PBS, followed by fixation with 100 μL of methanol for 15 min, after which the methanol was aspirated and the plate was air-dried, then the biofilms were stained with 100 μL of 1% crystal violet (G1062, Solarbio, Beijing, China) for 5 min and dried at 37 °C, and subsequently the crystal violet was solubilized with 100 μL of 33% glacial acetic acid (v/v) at 37 °C for 30 min, and the OD value of each well was measured at 570 nm using a microplate reader (Tecan Infinite M200 PRO.

$$\begin{array}{l} \text{Biofilm inhibition rate}\\ =\frac{\text{absorbance of control group}-\text{absorbance of experimental group}}{\text{absorbance of control group}}\times 100\% \end{array}$$

For quantification of biofilm live cells. After 24 h of incubation using the mentioned procedure. The attached biofilm was released using PBS, and the resultant biofilm was dispersed with an ultrasonicator for 10 min. The suspension passed through serial dilution to 10^5^ cells/mL, 100 μL cells distributed on an LB agar plate and then incubated at 37 °C for 24 h. Subsequently, the CFUs were determined by colony counting.

### 2.7 Scanning electron microscopy (SEM) analysis of biofilm

From a 24-h culture in 24-well microtiter plates, the biofilms (cultivated on glass slides) were carefully washed thrice with PBS, remaining with 2.5% glutaraldehyde (P1126, Solarbio, Beijing, China) for 4 h. The samples were rinsed twice with PBS buffer, dried, gold-plated, and observed using a field emission scanning electron microscope (Hitachi, SU-8220, Japan).

### 2.8 *In vivo* models of atopic dermatitis

Twenty four specific pathogen-free (SPF) female BALB/c mice, aged 6 weeks, weighing between 18 ± 2 g, were purchased from Beijing Si Pei Fu Laboratory Animal Technology Co. Ltd. mice were randomly divided into four groups (*n* = 6), normal control group (NC), model group (MD), positive control mupirocin group (MUP), and DY299-CFS treatment group (299-CFS). AD injury was induced using 2,4-dinitrochlorobenzene (DNCB) + MRSA in all mice except the NC group. On day 1, 200 μL of 1% DNCB solution was applied to the depilated area to sensitize, and the same method was reinforced once on days 3 and 5. On day 6, 300 μL of MRSA suspension at a concentration of 1 × 10^6^ CFU/mL was applied to the hair removal area on the back of the mouse to colonize and further induce dermatitis. Thereafter, 0.1% DNCB (200 μL) was used on the dorsal skin three times a week for 3 weeks to maintain AD-like lesions. From day 7, mice in the 299-CFS and MUP groups were treated with DY299-CFS-CFS24 (200 μL) and MUP (200 μL, 2% w/w) for 21 days, respectively, and no treatment was done in the NC and MD groups. All animals were executed, and dorsal skin and serum samples were collected. Samples were collected and stored at −80 °C.

### 2.9 Evaluation of dermatitis severity

The severity of dorsal skin dermatitis was evaluated by scoring of dermatitis (SCORAD), which represents an established method to assess the signs of AD; the skin damage of the backs of the mice in each group was observed, including erythema, edema, desquamation, and scabbing, and was graded as none (0 points), mild (1 point), moderate (2 points), severe (3 points), and very severe (4 points) (Tang et al., 2022).

### 2.10 Determination of stratum corneum hydration (SCH)

BALB/C Mice (NC group, *n* = 6; MD group, *n* = 6; 299-CFS, *n* = 6; MUP, *n* = 6) were used in the SCH experiment. On the last day of treatment, the animals were placed in a prone position, and stratum corneum hydration (SCH) was measured on the glabrous dorsal skin using a dermatometer (Osay, China) with the probe held perpendicular to the skin surface for 30 s.

### 2.11 Hematoxylin and eosin (H&E) staining

Dorsal skin was fixed in 4% paraformaldehyde and embedded in paraffin blocks. The blocks were cut into 4.5 μm-thick sections and stained with hematoxylin and eosin (G1120, Solarbio, Beijing, China) for pathological scoring. The pathological scores were calculated by summing up the scores for keratosis degree of epidermis, infiltration of cells, fibrous hyperplasia, and hyperplasia of skin tissue on the following scale: 0 (none), 0.5 (mild), 1 (mild-to-moderate), 2 (moderate), and 3-4 points (severe). Three sections (*n* = 3) from individual mice were examined blindly with 50× magnification by the pathologist, and the summed scores were compared between each group. In addition, for evaluating mast cell infiltration in the skin tissue, toluidine blue (Solarbio, China) staining was used. The number of dermal mast cells was calculated at 200× magnification in three fields.

### 2.12 Enzyme-linked immunosorbent assay (ELISA)

Serum levels of IgE, TNF-α, IFN-γ, IL-13, IL-17, and IL-1β were determined using commercial ELISA kits (UpingBio, China) according to the manufacturer’s protocols. Briefly, 50 μL of standards or serum samples were added to designated wells, followed by 100 μL of biotin-conjugated detection antibody and incubation at 37 °C for 60 min. After washing, 100 μL of HRP-conjugated avidin was added and incubated for 20 min at 37 °C. Following another wash, 100 μL of TMB substrate was added and incubated for 15 min at 37 °C in the dark. The reaction was terminated with 50 μL of stop solution, and absorbance was measured at 450 nm. A standard curve was generated using a four-parameter logistic (4-PL) regression model, and sample concentrations were calculated from the curve.

### 2.13 RNA-Seq and quantitative RT-PCR analysis

Total RNA was extracted from skin tissue and enriched for mRNA using Oligo (dT) magnetic beads. The RNA was fragmented to an approximate length of 300 bp using ion interruption. Using the fragmented RNA as a template, the first-strand cDNA was synthesized with hexamer random primers and reverse transcriptase, followed by the synthesis of the second-strand cDNA. After library construction, the library fragments were enriched by PCR amplification, and the RNA-seq library was constructed based on the fragment size. Following library construction, the quality of the library was assessed using an Agilent 2,100 Bioanalyzer to determine the total concentration and effective concentration of the library. After passing the quality control, the libraries containing different index sequences were mixed in proportion according to the effective concentration and the required sequencing data output. The mixed libraries were then diluted and alkali-denatured to form single-strand libraries. Subsequently, second-generation sequencing technology (Next-Generation Sequencing, NGS) was employed for paired-end sequencing on the Illumina sequencing platform (Anoroad Gene Technology Co., Ltd.).

Total RNA was extracted from skin tissue using the TRIzol (Invitrogen) method. The concentration and purity of the RNA were determined using a spectrophotometer, and the RNA was immediately stored in a −80 °C freezer. The RNA was reverse-transcribed into cDNA. The amount of cDNA was quantified using 5 × TransScript All-in-one Super Mix for qPCR (Q20926, Transgen, Beijing, China) with primers 590 & 591 for *Ccl3*, 592 & 593 for *Ccl4*, 596 & 597 for *Cxcl2*, 600 & 601 for *Mmp3*, 606 & 607 for *Lamc2*, 608 & 609 for *Slc2a1*, 614 & 615 for *Il17ra*, 616 & 617 for *Traf6*, 628 & 629 for *β-actin*. The reaction system included 10 μL of 2 × TransStart Top Green qPCR Super Mix, 8.3 μL of ddH₂O, 0.5 μL of cDNA, 0.4 μL of each forward and reverse primer, and 0.4 μL of Passive Reference Dye (50 ×) (AQ602, Transgen, Beijing, China). The RT-qPCR reaction conditions were as follows: initial denaturation at 94 °C for 30 s, followed by 40 cycles of amplification (denaturation at 94 °C for 5 s and annealing/extension at 60 °C for 30 s). β-actin was used as the internal reference gene, and the relative expression levels of the relevant genes were calculated using the 2^−ΔCt^ method. The primer sequences are detailed in Table 1.

**Table 1:** RT-PCR primer information.

Primer no.	Sequence
590	tgt acc atg aca ctc tgc aac
591	caa cga tga att ggc gtg gaa
592	ttc ctg ctg ttt ctc tta cac ct
593	ctg tct gcc tct tt tgg tca g
596	cca acc acc agg cta cag g
597	gcg tca cac tca agc tct g
600	ggc ctg gaa cag tct tgg c
601	tgt cca tcg ttc atc atc gtc a
606	cag agt tca gga tac gag cag ac
607	gtc tgc caa tct tgt agc ctc c
608	gct tct cca act gga cct caa ac
609	acg agg agc acc gtg aag atg a
614	ctg tat gac ctg gag gct ttc tg
615	cga gta gac gat cca gac ctt c
616	ttt ccc tga cgg taa agt gcc c
617	acc tgg cac ttc tgg aaa gga c
626	act cct acg gga ggc agc ag
627	gga cta chv ggg twt cta at
628	tgc tgt ccc tgt atg cct ct
629	atg tca cgc acg att tcc

### 2.14 LC–MS/MS analysis cell-free supernatants

A 1 mL volume of the supernatant from DY299-CFS was subjected to freeze-drying using a lyophilizer. Subsequently, 100 μL of an 80% methanol solution was introduced into the dried sample. The mixture was thoroughly vortexed to ensure homogeneity and then incubated in an ice bath for 5 min to stabilize the sample components. Following incubation, the sample was centrifuged at 15000g for 15 min at 4 °C to separate the supernatant from any precipitates or particulate matter. A defined volume of the resulting supernatant was subsequently diluted with mass spectrometry-grade water to achieve a final methanol concentration of 53%. This diluted sample was then subjected to a second centrifugation at 15000g for 15 min at 4 °C to further remove any residual particulates. The final supernatant was carefully collected and prepared for analysis via liquid chromatography-mass spectrometry (LC–MS).

The LC–MS analysis was conducted under the following conditions: Chromatographic separation was achieved using a Hypersil Gold C18 column. The injection volume was precisely controlled at 5 μL, while the flow rate was maintained at 0.2 mL/min. Gradient elution was employed to optimize the separation of analytes, with mobile phase A consisting of a 0.1% formic acid solution and mobile phase B comprising methanol. Throughout the entire analysis, all samples were stored in the autosampler at a constant temperature of 4 °C to ensure sample stability and consistency. To monitor and assess the stability of the analytical system and the reliability of the experimental data, quality control (QC) samples were periodically inserted into the sample queue. The LC–MS analysis was performed by Novogene Co., Ltd., located in Beijing.

Differential metabolites between groups were identified using orthogonal partial least-squares discriminant analysis (OPLS-DA). The variable importance in projection (VIP) values were calculated to evaluate the contribution of each metabolite to the classification model. Metabolites with VIP > 1, fold change (FC) ≥ 1.2, and *p* < 0.05 (determined by a two-tailed Student’s *t*-test) were considered as significantly differential metabolites. Among these, the top 20 metabolites with the highest FC values were selected for subsequent network pharmacology analysis.

### 2.15 Network analysis

Firstly, the SMILES IDs of the compounds were obtained through the PubChem database^1^. These SMILES IDs were then imported into the SwissTargetPrediction database^2^ to obtain the corresponding compound targets. Targets with a probability score greater than 0 were selected and included. Secondly, using “Atopic dermatitis” as the keyword, human gene searches were conducted in the GeneCards database^3^, the NCBI Gene database^4^, and the CTD database^5^. The intersection of drug targets and disease targets was taken, and the intersecting targets were imported into the STRING platform^6^, with the confidence level set at 0.4, to obtain protein–protein interaction (PPI) network data. This data was then imported into Cytoscape 3.8.0 software for visualization processing. The common targets of drugs and diseases were subjected to Gene Ontology (GO) enrichment analysis, including biological process (BP), molecular function (MF), and cellular component (CC), as well as KEGG pathway enrichment. Items with a corrected *p*-value of less than 0.05 were screened out. Using R 4.1.2 software, the packages clusterProfiler, enrichplot, and ggplot2 were installed and cited to create bubble charts. The network files of components-diseases-pathways-targets were imported into Cytoscape 3.8.0 to draw pathway network diagrams, more intuitively demonstrating the multi-component and multi-target characteristics of the active components of DY299-CFS in the treatment of diseases.

### 2.16 Statistical analysis

Data processing was undertaken with GraphPad Prism (9.5.1) software (La Jolla, United States). Continuous variables are described by mean ± standard deviation (mean ± SD). For independent samples, a two-tailed Student’s *t*-test was used for two-group comparisons, while multiple groups were compared with one-way analysis of variance (ANOVA) followed by Tukey’s post-hoc test for pairwise comparisons. The results were considered statistically significant when the *p*-value was less than 0.05.

## 3 Results

### 3.1 Isolation of anti-biofilm bacteria

A total of 34 bacterial isolates were cultured and purified from Alshan surface water samples through standardized isolation protocols. The anti-biofilm potential of these producer strains was systematically assessed using the crystal violet semi-quantitative adhesion method testing. Anti-biofilm spectra were determined through comprehensive evaluations against Methicillin-Resistant *Staphylococcus aureus* strain (USA300). Notably, strain *Bacillus halotolerans* DY299 (cell-free culture supernatant) exhibited highly anti-biofilm activity against MRSA (Table 2).

**Table 2:** The biofilm inhibitory effect of cell-free supernatant of DY292-DY303 against the MRSA.

Strain	Inhibition	Identified species
DY292	7.11%	*Bacillus* sp. strain
DY293	29.14%	Bacillus sp.
DY294	2.45%	*Bacillus megaterium* strain
DY295	15.94%	*Priestia aryabhattai* strain
DY296	0%	*Virgibacillus olivae* strain
DY297	0%	*Bacillus subtilis* strain
DY298	6.08%	Bacillus sp.
DY299	50.93%	*Bacillus halotolerans* strain
DY300	23.07%	*Bacillus swezeyi* strain
DY301	46.56%	*Bacillus swezeyi* strain
DY302	45.19%	Bacillus sp.
DY303	11.61%	*Bacillus subtilis* strain

Inhibition (%) = ((OD_570_ (control) − OD_570_ (treated) × 100)/OD_570_ (control)).

### 3.2 Morphological observation and biological characteristics of *Bacillus halotolerans*-DY299

Morphological characterization of strain *Bacillus halotolerans* DY299 revealed that colonies grown on LB agar plates at 37 °C for 24 h were white, circular, 2-3 mm in diameter, viscous, raised, with smooth and moist surfaces, and intact margins (Figure 1A). Gram staining showed purple-colored, short, and thick cells, confirming its classification as a Gram-positive bacterium (Figure 1B). For phylogenetic identification, the 16S rRNA gene sequence of strain *Bacillus halotolerans* DY299 was analyzed via BLAST comparison. Neighbor-joining analysis using MEGA (v.11) revealed that *Bacillus halotolerans* DY299 shared 99% sequence similarity with *Bacillus halotolerans* strain ZSTM2175/A. Homology between *Bacillus halotolerans* strain DY299 and other existing whole-genome datasets was evaluated using Average Nucleotide Identity (ANI) analysis with fastANI^7^. DY299 was found to share the highest homology with *Bacillus halotolerans* NRRL B-41618. Therefore, the strain was belongs to the phylum *Firmicutes*, class *Bacilli*, order *Bacillales*,genus *Bacillus* and strain *Bacillus halotolerans* (Figure 1C, Table 3).

**Figure 1:**
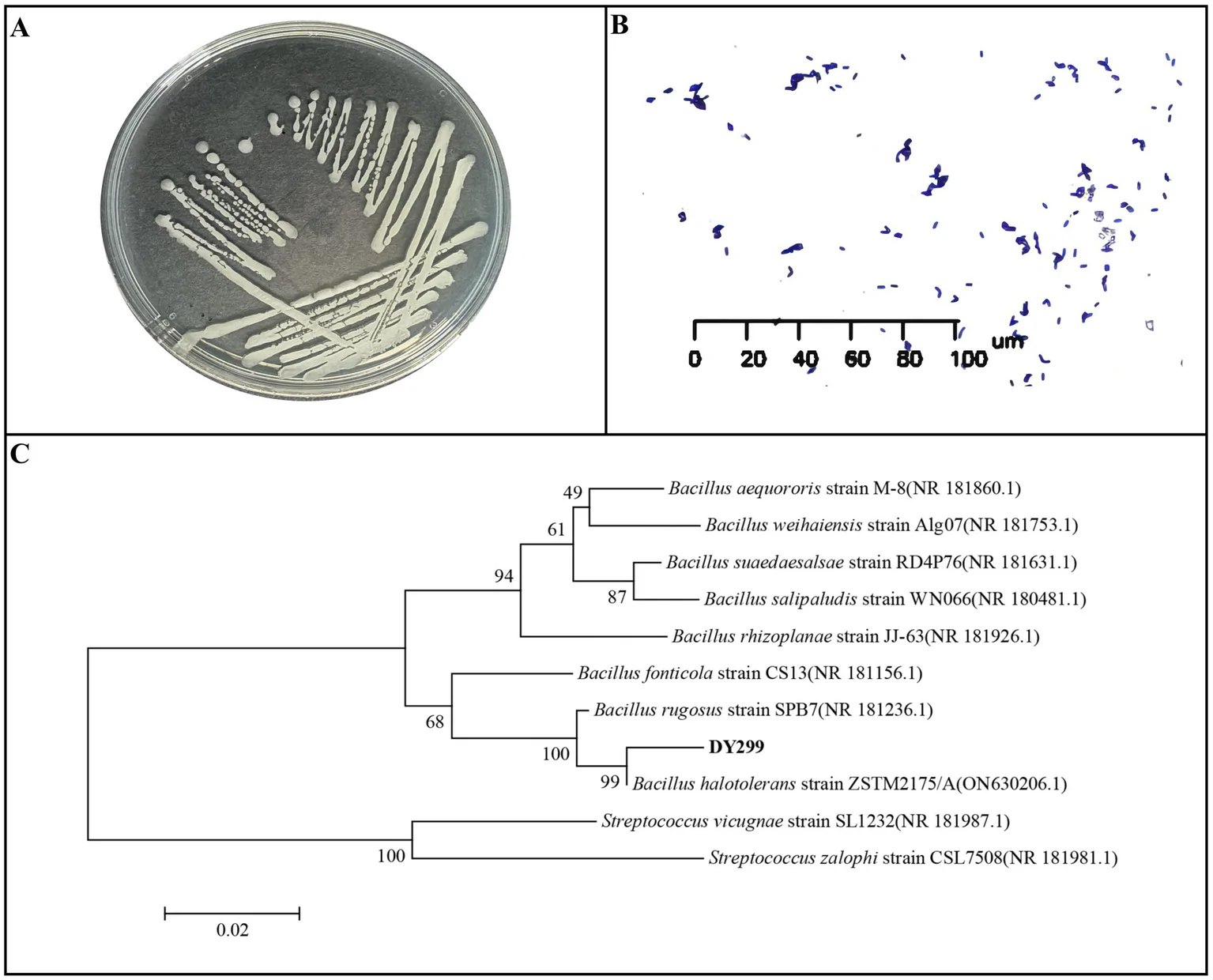
Morphological observation and molecular identification of strain *Bacillus halotolerans* DY299. **(A)** Bacteria cultured at 37 °C for 24 h showed smooth glossy and rod-shaped colonies, **(B)** Microscopic image of *Bacillus halotolerans* DY299 with Gram stain, **(C)** Phylogenetic tree of *Bacillus halotolerans* DY299.

**Table 3:** Results of comparing the genome of *Bacillus halotolerans* DY299 with existing whole-genome data.

Strain	Homology (%)
*Bacillus halotolerans* NRRL B-41618	97.73
*Bacillus halotolerans* III-1	97.69
*Bacillus halotolerans* FJAT-2398	97.64
*Bacillus halotolerans* ATCC 25096	97.64
*Bacillus rugosus* SPB7	87.64
*Bacillus fonticola* CS13	67.66

The growth characteristics of *Bacillus halotolerans-*DY299 were systematically investigated. Temperature tolerance assays demonstrated that strain *Bacillus halotolerans-*DY299 exhibited optimal growth at 37 °C within a broad range of 10–45 °C (Figure 2A). Salinity adaptation analysis revealed a halotolerant phenotype, with viable growth observed across 2–6% (w/v) NaCl concentrations, and the OD value reaches its maximum at 6% (Figure 2B). Similarly, pH tolerance profiling identified a growth range of pH 4–12, with neutral pH 7.0 as the optimum condition (Figure 2C). To correlate environmental parameters with population dynamics, a 24-h growth kinetic study was conducted under defined conditions (6% NaCl, pH 7.0, 37 °C), yielding a sigmoidal growth curve characteristic of *Bacillus halotolerans-*DY299, and it was in the logarithmic growth phase within 12 h (Figure 2D).

**Figure 2:**
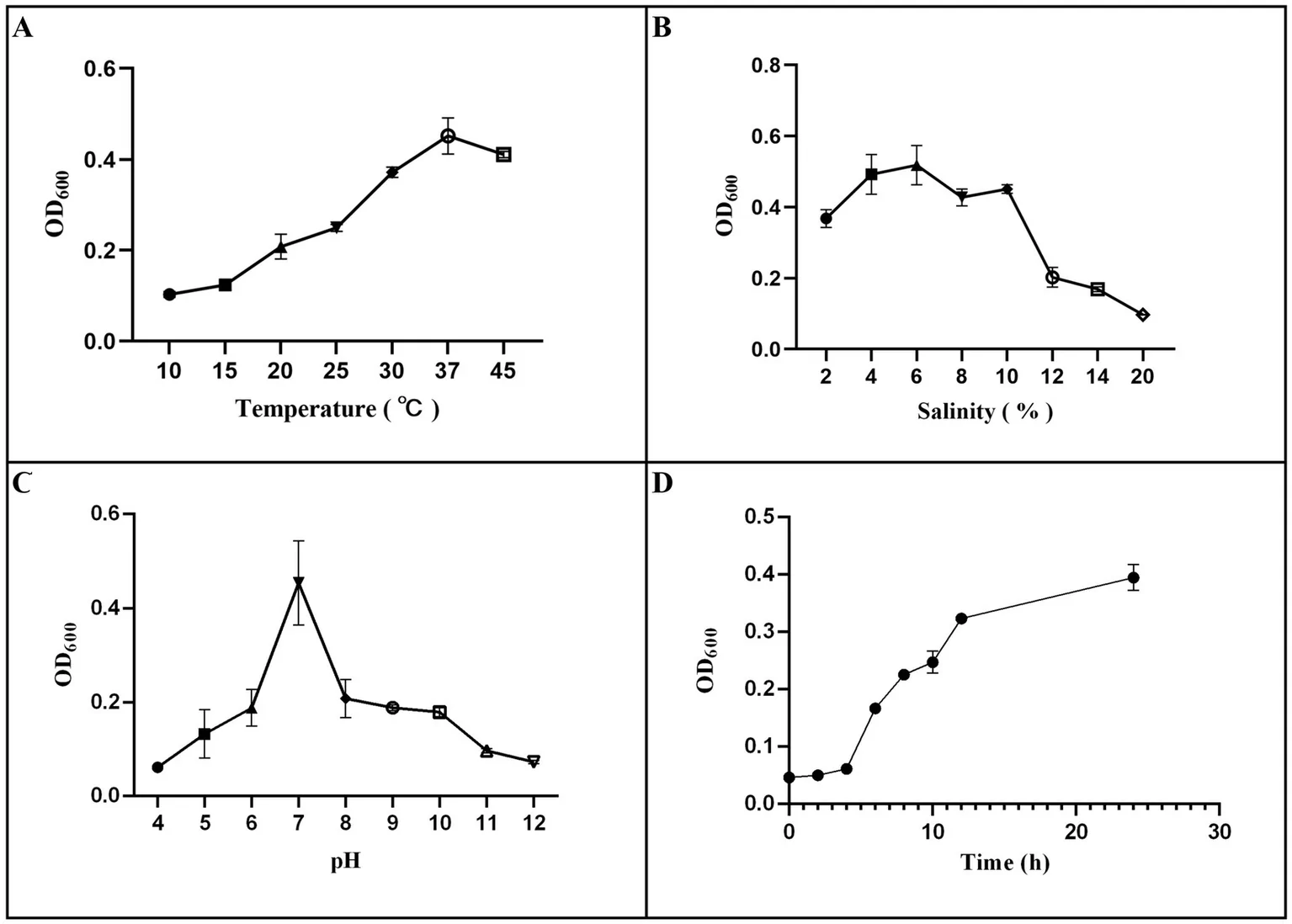
Growth characteristics of *Bacillus halotolerans* DY299. **(A)** Growth occurs at temperature ranging from 10 to 45 °C, **(B)** Growth occurs at ranging from 0 to 20% NaCl (w/v), **(C)** Growth occurs at pH ranging from 4 to 12, **(D)** The growth curve of the strain DY299 was in logarithmic phase within 12 h.

### 3.3 DY299-CFS inhibits the MRSA growth and formation of MRSA biofilm

The inhibitory effect of different concentration of DY299-CFS the growth of MRSA was shown in Figure 3A. The dynamic antibacterial curve shows that the growth rate tends to level off between 12 and 24 h. The OD (600) values of the MRSA growth curves in the CFS-25 and CFS-50 treatment groups were significantly lower than those in the control group, indicating that bacterial growth was significantly inhibited. The results suggest that DY299-CFS can continuously inhibit the growth of MRSA.

**Figure 3:**
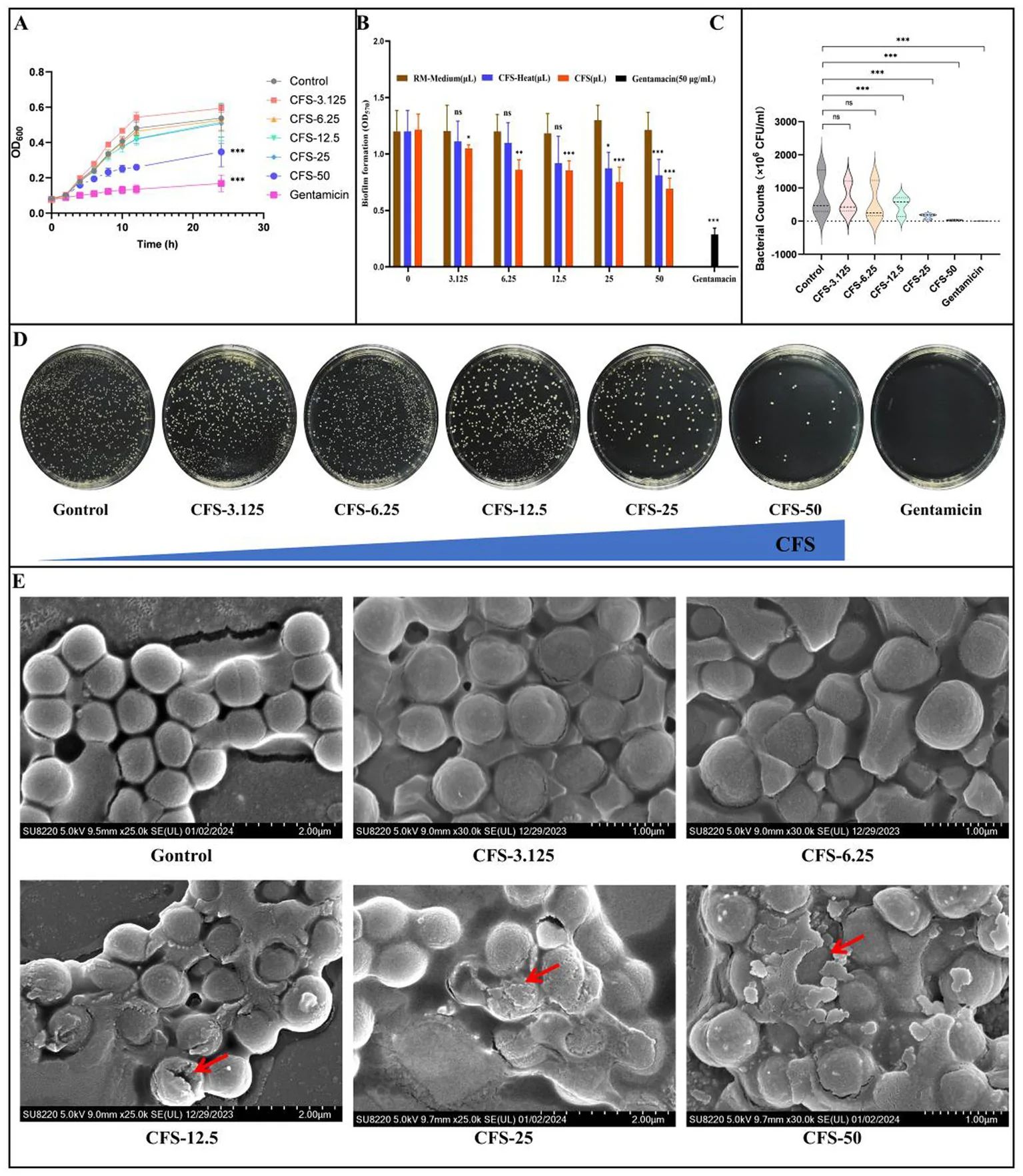
Anti-MRSA biofilm activity of DY299-CFS **(A)** MRSA growth curve with different concentration of DY299-CFS **(B)** Effect of DY299-CFSs on biofilm formation by MRSA strain. The CFS and CFS-Heat groups were paired with an equal volume of RM medium as the control, while the Gentamicin group used 0 volume of RM medium as the absolute control **(C,D)** CFU counting of the biofilm cells after treatment with different concentration of the DY299-CFS **(E)** SEM images of MRSA with different concentration DY299-CFS for 24 h. Data are expressed as means ± SDs. Significantly different means are indicated with asterisks (ns: not significant, *p < 0.05, **p < 0.01, and ***p < 0.001).

The crystal violet staining method was employed to evaluate the inhibitory effects of various concentrations of DY299-CFS on MRSA biofilm formation. The results showed that the OD (570) values of the DY299-CFS treatment groups (concentrations ranging from CFS-3.125 to CFS-50) were significantly lower than those of the control group (equal volume of RM medium), indicating that DY299-CFS possesses significant anti-biofilm activity against MRSA (Figure 3B). Furthermore, comparative analysis revealed that the heat-treated DY299-CFS group exhibited different biofilm inhibition effects compared to the active DY299-CFS group at equivalent concentrations. At volumes of 50 μL and 25 μL, the heat-treated group retained significant inhibitory effects comparable to the untreated active group; however, at 12.5 μL and 6.125 μL, it failed to demonstrate the same significant biofilm inhibition as the active DY299-CFS group, suggesting that heat treatment partially compromised its anti-biofilm efficacy. Meanwhile, the RM broth medium control group (without DY299) showed no significant inhibitory effect on biofilm formation compared to the untreated control group, excluding the possibility that residual culture medium components were responsible for the observed activity. Collectively, these findings indicate that the bioactive substances responsible for biofilm inhibition include both heat-stable small-molecule metabolites produced by DY299-CFS and heat-labile molecules (such as peptides), and that the activity is unrelated to medium components.

The colony forming units (CFU) of viable bacteria within the biofilms was counted after treatment with different concentrations of DY299-CFS. Compared with the control group, the number of viable bacteria in the CFS-12.5, CFS-25, and CFS-50 treatment groups was significantly reduced, indicating that DY299-CFS can kill viable bacteria within MRSA biofilms. The ability of DY299-CFS to inhibit viable bacteria within biofilms is concentration-dependent (Figures 3C,D).

Scanning electron microscopy was used to observe the effects of DY299-CFS on the morphological structure of MRSA. The MRSA cells in the control group, which were not treated with DY299-CFS, had typical cellular structures. The bacterial cells were round, with smooth surfaces, no damage to the cell surface, intact structures, and good growth without aggregation or shrinkage. In contrast, the cell walls and membranes of MRSA treated with different concentrations of DY299-CFS (12.5–50) were damaged, and the bacterial cells were deformed and lysed, with loss of cell integrity and overall structural collapse. These findings indicate that DY299-CFS can effectively damage the structure of MRSA, causing leakage of cellular contents (Figure 3E).

### 3.4 DY299-CFS attenuated the MRSA-induced atopic dermatitis in mice

DY299-CFS significantly ameliorated the pathological phenotypes of MRSA-induced atopic dermatitis (AD) in a murine model and demonstrated multidimensional therapeutic effects (Figure 4A). In this experiment, the mupirocin (MUP) group was designated as the positive pharmacological control group. As an infectious agent, MRSA significantly exacerbates tissue dryness and skin damage in patients with AD through mechanisms involving barrier disruption, amplification of inflammatory responses, and impairment of tissue repair. In a murine model of AD, treatment with DY299-CFS for 3 weeks resulted in a significant increase in skin water content (Figure 4B) and a marked reduction in skin dermatitis scores (Figure 4C). Quantitative analysis of MRSA colonization in skin tissues revealed that compared to normal control mice, the AD group exhibited elevated MRSA counts, confirming successful MRSA colonization on the skin. Notably, the DY299-CFS treatment group demonstrated a significant reduction in MRSA counts compared to the AD group, indicating the antimicrobial efficacy of DY299-CFS against MRSA colonization on the skin (Figure 4D).

**Figure 4:**
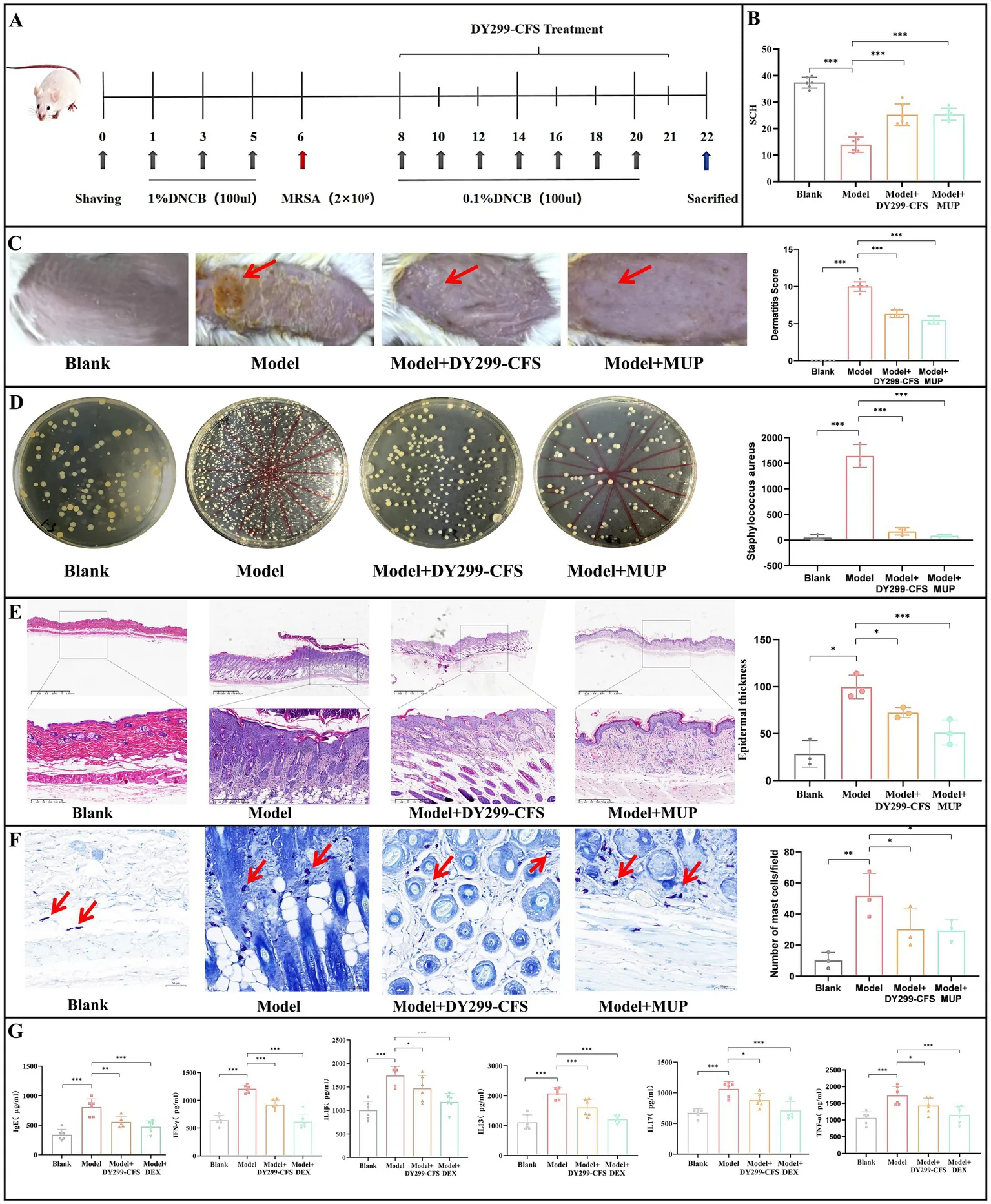
Experimental setting of the *in vivo* studies and results on DY299-CFS treatment of MRSA-induced AD in the experimental mice. **(A)** Schematic illustration of the *in vivo* experimental procedures. **(B)** Data of the SCH change in model group and DY299-CFS treatments. **(C)** *In vivo* observation a representative photographs of skin lesions infected with MRSA and treatment with DY299-CFS. **(D)** *In vivo* antibacterial efficacy test images for each group and the count of MRSA colonies. **(E)** Histological analysis of the hydrogels a Representative histological sections images of MRSA-infected skin lesions with H&E stain. **(F)** The number of mast cells in control and experimental groups. **(G)** Serum levels of IgE, IL-17, IL-13, IL-1β, IFN-γ, and TNF-α in each group were measured by ELISA. The model group showed significantly elevated levels of these inflammatory cytokines and immunoglobulin compared to the control group, while treatment with DY299-CFS markedly reduced their concentration (*p < 0.05, **p < 0.01, ***p < 0.001 vs. model group, and *n* = 6).

Histopathological examination of mouse skin tissues revealed distinct structural differences among the groups. The control group exhibited intact skin architecture with well-defined epidermal layers and no signs of epidermal thickening or inflammatory cell infiltration. In contrast, the AD group displayed hyperkeratosis, thickening of the stratum corneum, hypertrophy of the spinous layer, and papillary projections of the epidermis into the dermis. The dermis in the AD group was characterized by collagen fiber proliferation, capillary dilation and congestion, and marked inflammatory cell infiltration. DY299-CFS treatment effectively attenuated these pathological changes by inhibiting the increase in epidermal thickness and reducing inflammatory cell infiltration (Figure 4E).

Toluidine blue staining was employed to quantify and assess the degranulation status of mast cells in the skin tissues. The model group exhibited a significant infiltration of mast cells in the dermis of the dorsal skin lesions, with many cells in a degranulated state, resulting in a higher mast cell count compared to other groups. In contrast, the DY299-CFS intervention group showed a marked reduction in mast cell counts, highlighting the anti-inflammatory potential of *Bacillus halotolerans* DY299-CFS in modulating mast cell activity in AD-affected skin (Figure 4F). ELISA was employed to quantitatively analyze inflammatory immune factors in mouse serum. The serum levels of IgE, IL-17, IL-13, IL-1β, IFN-γ and TNF-α were significantly increased in the model group compared to the control group,whereas the group treated with *Bacillus* DY299-CFS showed a significant decrease (Figure 4G).

### 3.5 DY299-CFS inhibits the expression of MRSA-induced interleukin-17 cytokines and chemokines in an atopic dermatitis model

To further explore the effects of DY299-CFS on the gene expression of skin tissues in the MRSA-induced Atopic Dermatitis model, we conducted transcriptomic analysis on the skin tissues from the blank group, model group, and DY299-CFS group. By creating volcano plots of differentially expressed genes, we first identified 505 differentially expressed genes in the comparison between the blank group and the model group, of which 46 were upregulated and 459 were downregulated (Figure 5A). In the comparison between the model group and the DY299-CFS treatment group, we found 451 differentially expressed genes, with 273 upregulated and 178 downregulated (Figure 5B). Subsequently, we compared these two sets of differentially expressed genes and identified 51 commonly differentially expressed genes (Figure 5C). We then performed GO analysis (Figure 5D) and KEGG analysis (Figure 5E) on these common differentially expressed genes. The results of the GO and KEGG analyses showed that these differentially expressed genes were mainly involved in the Interleukin-17 (IL-17) and Chemokines signaling pathways. *S. aureus* can not only colonize the surface of the skin, but also penetrate the dermis, where it directly contacts immune cells and stimulates the production of pro-inflammatory cytokines. *S. aureus* produces a series of potent virulence factors that induce the generation of IL-17-producing cells, which are the mediators of the skin’s inflammatory response to *S. aureus* (Ogonowska et al., 2021). Therefore, we further validated the expression of genes in the IL-17 signaling pathway and the chemokines signaling pathway genes through RT-PCR (*Ccl3*, *Ccl4*, *Cxcl2*, *Lamc2*, *IL17-ra* and *Traf-6*) (Figures 6A–F).

**Figure 5:**
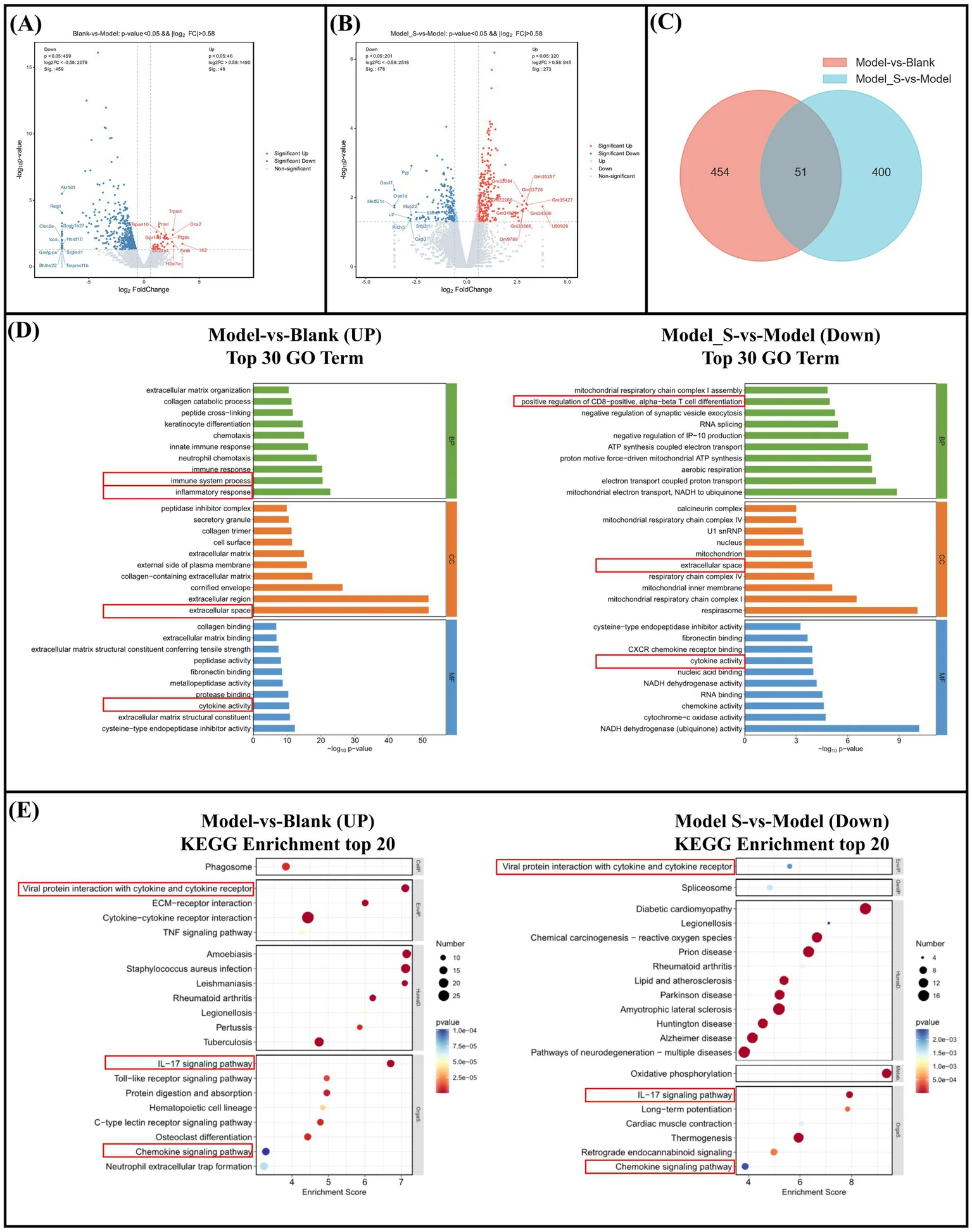
RNA-Seq analysis reveals genes and pathways targeted by DY299-CFS. **(A)** Volcano plot of mRNAs expression in control (Blank) group and model group skin tissue of mouse. **(B)** Volcano plot of mRNAs expression in DY299-CFS treated group (Model-S) and model group skin tissue of mouse. **(C)** Venn diagram of the genes in Model vs. Blank and Model-S vs. Model. **(D)** GO enrichment analysis of differentially expressed genes (DEGs) in Model vs. Blank (up-regulated) and Model-S vs. Model (down-regulated). **(E)** Barplot showing the KEGG enrichment of DEGs in Model vs. Blank (up-regulated) and Model-S vs. Model (down-regulated). Different colors indicate different adjusted *p*-values (*p* < 0.05).

**Figure 6:**
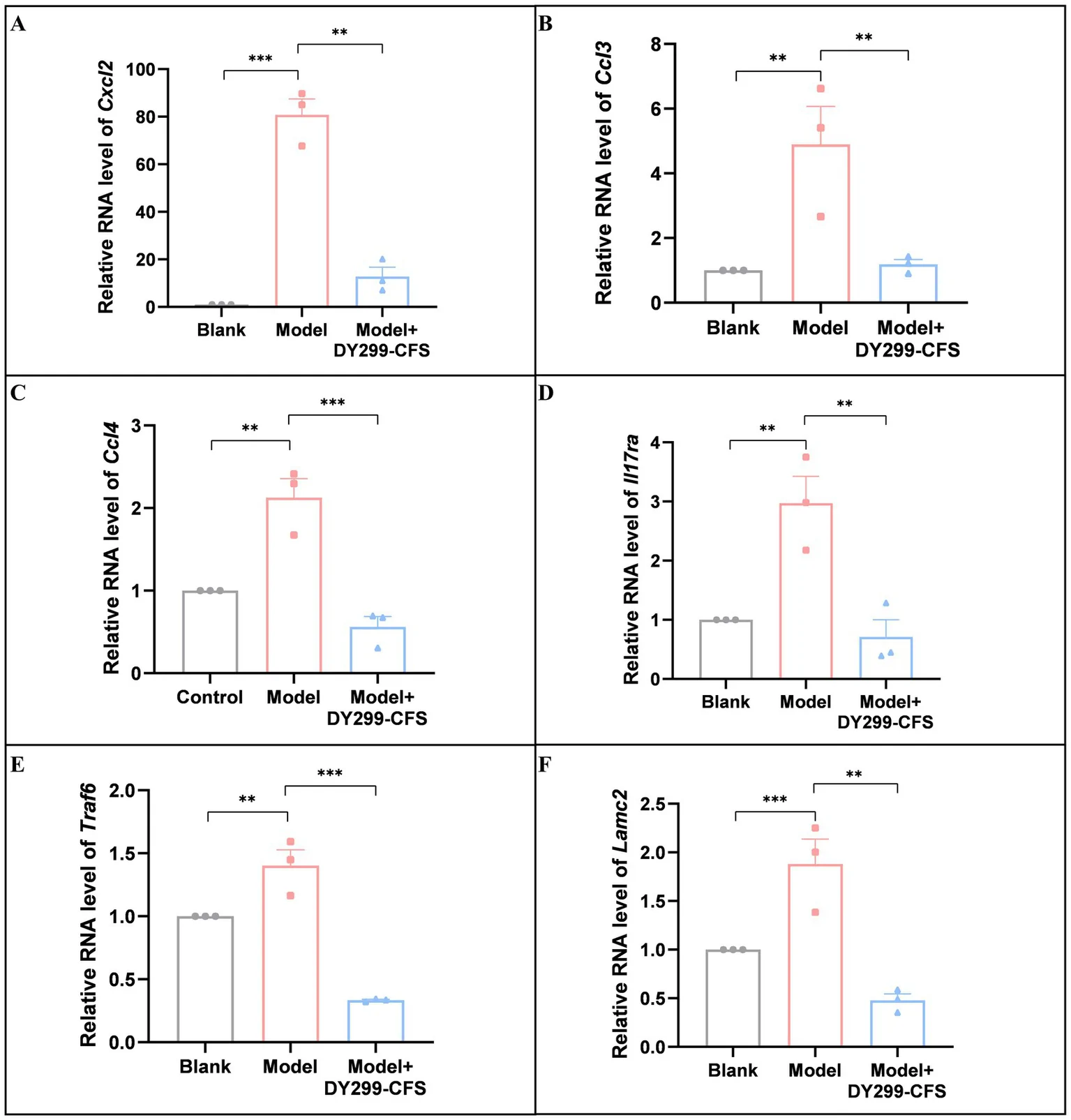
Effects of DY299-CFS on the expression of IL-17 signaling pathway and chemokine-related genes. **(A)** Cxcl2, **(B)** Ccl3, **(C)** Ccl4, **(D)** Il17a, **(E)** Traf6, **(F)** Lamc2. mRNA levels were measured by RT-qPCR and normalized to β-actin. Data are expressed as means ± SDs (*n* = 3). Significantly different means are indicated with asterisks (*p < 0.05, **p < 0.01, ***p < 0.001 vs. Model group.

### 3.6 Whole genome sequencing and analysis secondary metabolites of DY299-CFSs

The sequencing of *Bacillus halotolerans* DY299 was conducted using a combination of second-generation Illumina and third-generation Nanopore sequencing methods. The results showed that the genome has a total length of 4,208,811 bp, with an average GC content of 43.75%. The genome consists of a single circular chromosome. A total of 4,462 coding genes were identified, with a combined length of 3,735,972 bp. Non-coding RNA analysis revealed that *Bacillus halotolerans* DY299 contains 86 tRNAs (Figure 7A).

**Figure 7:**
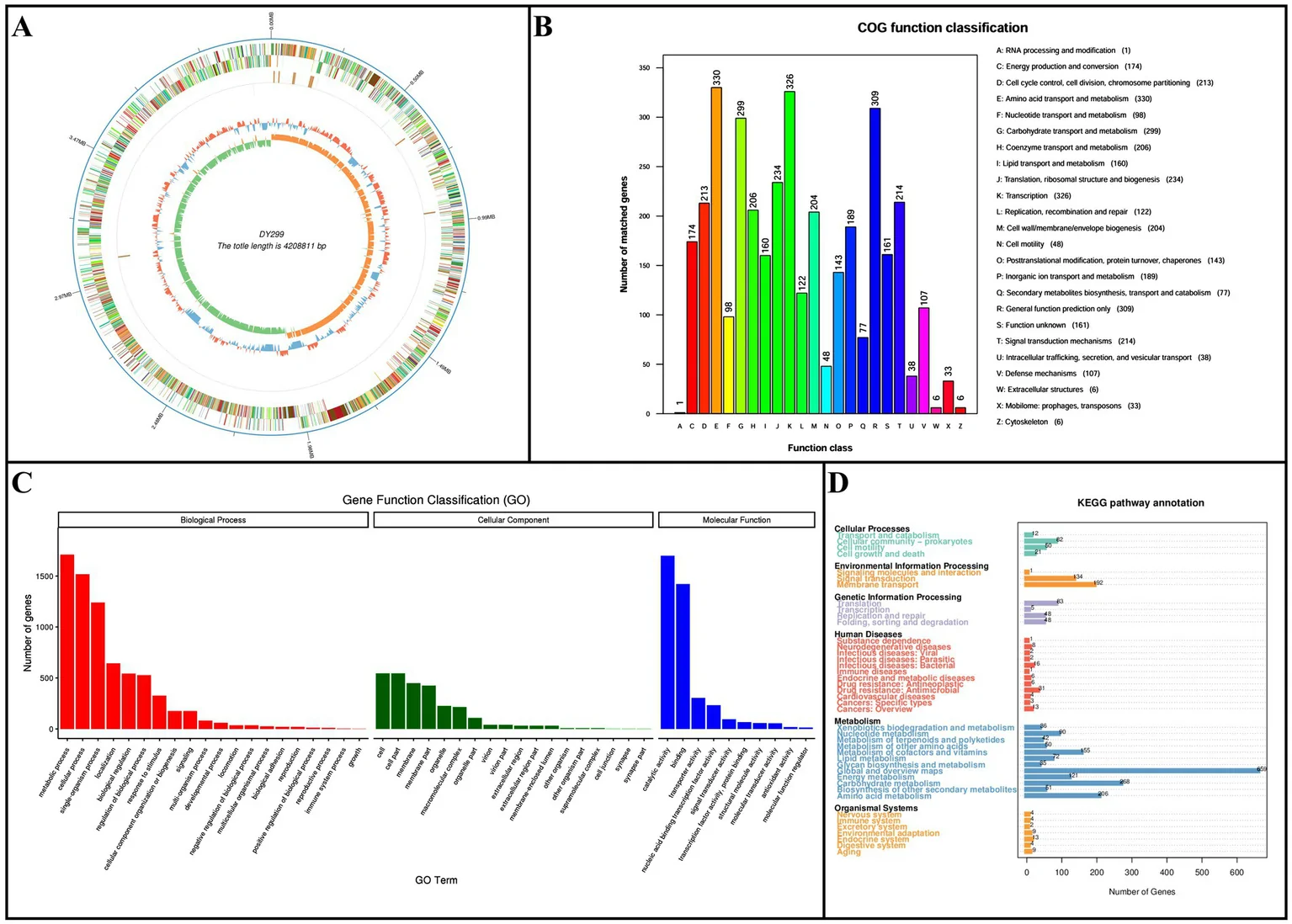
Whole-genome map and COG annotation of *Bacillus halotolerans* DY299. **(A)** Marked characteristics are shown from the outside to the center; coding sequence (CDS) on the forward strand, CDS on the reverse strand, tRNA, rRNA, guanine-cytosine (GC)-content, and GC skew. CDS genome consists of a single circular chromosome that is 4.2 Mb in size. **(B)** COG functional classification diagram; **(C)** GO classification functional statistics; **(D)** KEGG metabolic pathway classification diagram).

Analysis using the clusters of orthologous genes (COG) database found that the functional types of *Bacillus halotolerans* DY299 mainly include amino acid transport and metabolism, transcription, general function prediction only, carbohydrate transport and metabolism, and 24 other functional categories. A total of 3,698 coding genes in the *Bacillus halotolerans* DY299 genome were annotated to these functional categories, accounting for 82.87% of the total coding genes (Figure 7B).

The GO database comprises three major classifications: biological process, molecular function, and cellular component. In the first-level classification results, the number of genes annotated to biological processes was the highest, followed by molecular functions. Within biological processes, the number of genes enriched in metabolic process, cellular process, and single-organism process was significantly higher than in other categories. In molecular functions, 1,692 and 1,422 genes were annotated to catalytic activity and binding, respectively (Figure 7C).

KEGG analysis showed that in the first-level classification, the number of genes annotated to metabolism was the highest, with a total of 1,785 genes. Among them, Global and overview maps involved 659 genes, which was the most in all second-level classifications. The next highest were carbohydrate metabolism and amino acid metabolism categories, with 268 and 206 genes annotated, respectively. It is preliminarily inferred that these three metabolic pathways play an important role in the antibiofilm function of *Bacillus halotolerans* DY299 (Figure 7D).

To analyze the active components in the DY299*-*CFS, the CFS was analyzed by the LC–MS/MS assay. Figures 8A,B illustrate that we identified 116 compounds in the negative (NEG) electrospray ionization modes of UHPLC-QE-MS, and 328 in the positive (POS) electrospray ionization modes. A total of 444 chemical components were identified, with 9 types of chemicals classified as alkaloids and derivatives, benzenoids, lipids and lipid-like molecules, organ heterocyclic compounds and organic acids and derivatives being the primary chemical constituents of DY299-CFS (Figure 8C). Based on the criterion that metabolites with a VIP value greater than 1 in PLS-DA are considered to make significant contributions to the model, differential metabolites were screened using the following thresholds: log₂FC ≥ 1, VIP > 1 from OPLS-DA analysis, and *p* < 0.05 as determined by a two-tailed Student’s *t*-test. After applying these criteria, an additional filtering step was performed to exclude metabolites that could potentially originate from the culture medium, thereby retaining only those confirmed to be of bacterial origin. As a result, a total of 40 bacterial-derived differential metabolites were identified, of which 11 were significantly upregulated in negative ion mode and 29 in positive ion mode (as shown in Table 4).

**Figure 8:**
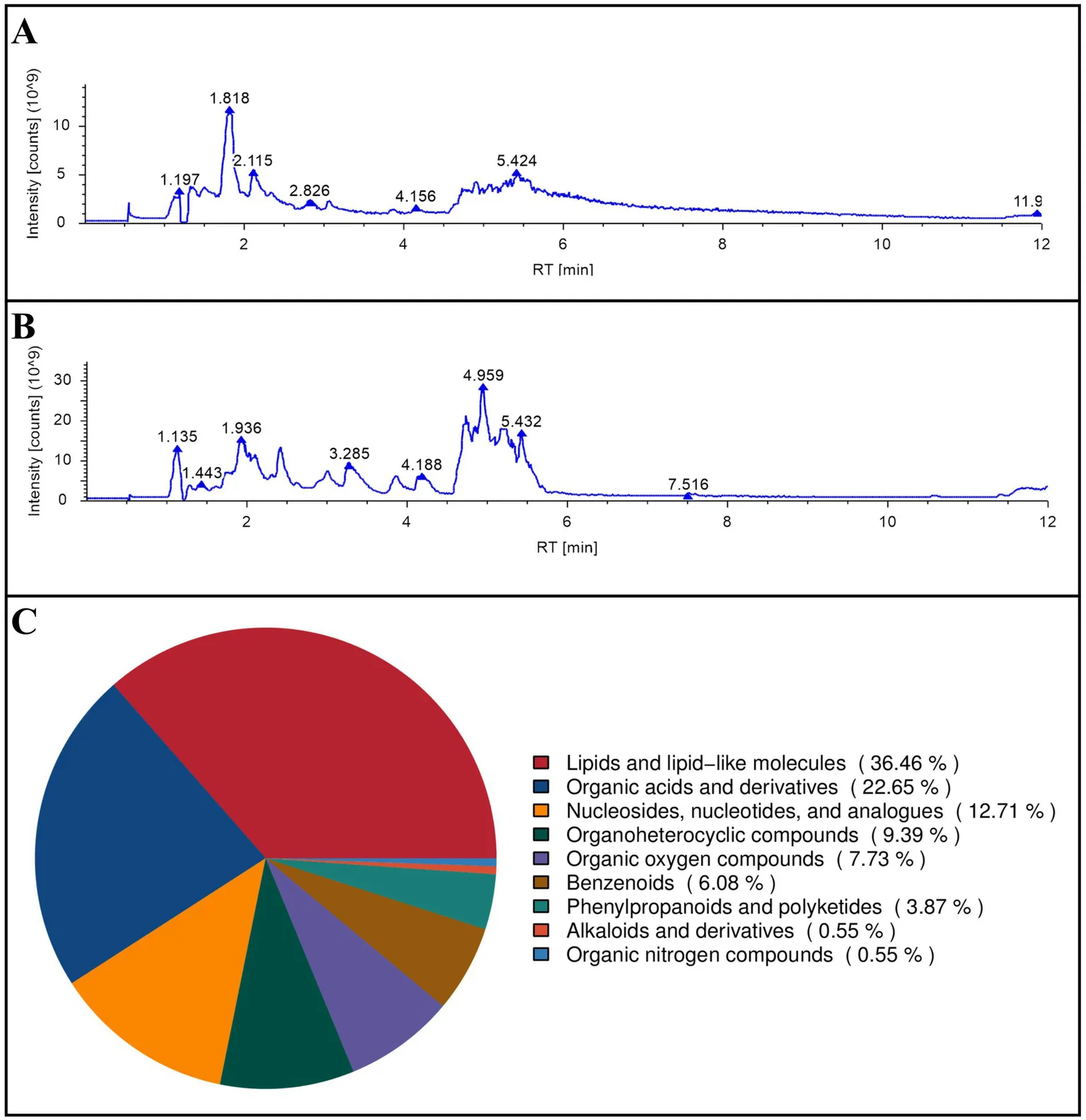
Analysis of components of DY299-CFS **(A)** UHPLC-QE-MS total ion chromatogram (TIC) of DY299-CFS under the negative (NEG) electrospray ionization mode **(B)** UHPLC-QE-MS TIC of DY299-CFS under the positive (POS) electrospray ionization mode **(C)** Classification of components of DY299-CFS detected by UHPLC-QE-MS analysis.

**Table 4:** The differentially abundant metabolites derived from bacteria in DY299-CFS.

No.	Name	RT [min]	m/z	log_2_FC	*p*-value	VIP
1	2-Hydroxyvaleric acid	5.330	117.05	3.17	0.000001	1.18
2	N-Acetylanthranilic acid	5.851	178.05	2.65	0.000002	1.18
3	cis-Aconitic acid	2.021	173.01	2.30	0.000011	1.18
4	2-Isopropylmalic acid	5.463	175.06	2.57	0.000035	1.18
5	6-Deoxy-D-glucose	1.545	373.14	1.59	0.000343	1.18
6	N6-Succinyl Adenosine	5.041	382.10	1.28	0.000441	1.18
7	Lipoic acid	2.639	205.03	4.04	0.000716	1.17
8	Asp-glu	1.594	243.06	1.95	0.013566	1.14
9	N-Acetyl-DL-glutamic acid	2.081	188.06	3.17	0.013786	1.12
10	Pseudouridine	1.586	243.06	2.00	0.013926	1.14
11	Citraconic acid	2.405	129.02	1.13	0.023149	1.12
12	Gly-Pro-Lys	1.381	301.19	5.34	0.000000	1.11
13	Thr-Gln-His	6.084	385.18	4.99	0.001090	1.11
14	His-Lys-Lys	6.443	412.27	4.73	0.000099	1.11
15	Thr-Met-Lys	1.604	379.20	4.36	0.000004	1.11
16	Phe-Met-His	1.517	439.14	4.33	0.000312	1.11
17	1-Methylguanine	2.440	166.07	3.97	0.000214	1.11
18	Agmatine	1.282	131.13	3.83	0.000002	1.11
19	Nicotinamide	1.461	123.06	3.63	0.000038	1.11
20	Gly-Tyr-Ala	4.894	310.14	3.26	0.000001	1.11
21	2-(2-hydroxy-3-methylbutanamido)-4-methylpentanoic acid	6.175	254.14	3.07	0.001076	1.11
22	1-Methyladenosine	4.806	282.12	3.05	0.000999	1.11
23	Arg-Pro-Lys	6.230	382.26	2.93	0.007449	1.09
24	Val-Asn-His	3.407	352.16	2.76	0.001730	1.09
25	Thr-Asn-Lys	2.435	344.19	2.66	0.000257	1.11
26	mesaconic acid	2.143	283.04	2.47	0.000001	1.11
27	Trp-Leu-His	4.729	455.24	2.38	0.004983	1.06
28	Glu-Met-Lys	4.158	407.20	2.37	0.006013	1.09
29	2,3,4-Trihydroxybenzoic acid	2.892	153.02	2.20	0.000808	1.11
30	N-Acetylornithine	1.465	175.11	2.03	0.000004	1.11
31	H-Gly-Pro-OH	3.846	367.16	1.97	0.001407	1.10
32	1-Methylguanosine	4.852	298.12	1.81	0.009027	1.06
33	Gly-Pro-His	2.814	292.15	1.70	0.002242	1.09
34	Gln-Pro-Lys	1.847	372.23	1.65	0.007313	1.09
35	(R)-3-Hydroxy myristic acid	5.784	262.24	1.40	0.045945	1.01
36	Pro-Asn-Lys	1.789	358.21	1.36	0.022170	1.06
37	Hypoxanthine	1.902	137.05	1.33	0.000001	1.11
38	Tyr-Met-Lys	4.789	423.21	1.24	0.000053	1.11
39	Lys-Met-His	1.526	415.21	1.17	0.001464	1.10
40	Asp-Pro-Lys	4.558	341.18	1.09	0.002164	1.07

RT (min), retention time (in minutes); *m*/*z*, mass-to-charge ratio; VIP, variable importance in the projection; FC, fold change.

To ensure transparency and reproducibility, detailed information is provided in Supplementary Table 1. This includes the names of the 40 metabolites, their predicted targets from SwissTargetPrediction, and the number of these targets that overlap with atopic dermatitis (AD)-related genes. Among the 40 metabolites, 36 were found to have at least one such overlapping target. There were 613 target genes related to the DY299-CFS components. There were 2,665 atopic dermatitis target genes obtained from the Gene Cards, NCBI, and CTD Databases. Using the Venn analysis, we identified 264 common target genes among the DY299-CFS and atopic dermatitis-related target genes (Figure 9A). In order to obtain more insight of the complex connections underlying components, diseases, and their respective targets, we created a network that covers the included components, therapeutic diseases, and its effect on the targets. We imported it into Cytoscape 3.8.0 to create a graphical illustration of the network (Figure 9B). GO and KEGG enrichment analyzes were conducted on these 264 target genes. The 264 of DY299-CFS ‘s target genes for atopic dermatitis treatment were introduced into String 11.5 to construct a PPI network, and the top 20 key genes were screened according to their degree value (HSP90AA1, STAT3, AKT1, SRC, HSP90AB1, EGFR, ESR1, MAPK1, PIK3CA, TNF, KRAS, PIK3CD, MAPK8, RELA, TLR4, PTPN11, CASP3, HIF1A, HDAC1 and TLR2). GO analysis indicated that these components significantly regulated the regulation of inflammatory response, and response to molecule of bacterial origin (Figure 9C). In the KEGG enrichment analysis, most of the target genes were significantly enriched in the pathogen infection, inflammatory signaling pathway, such as IL-17 signaling pathway (Figure 9D).

**Figure 9:**
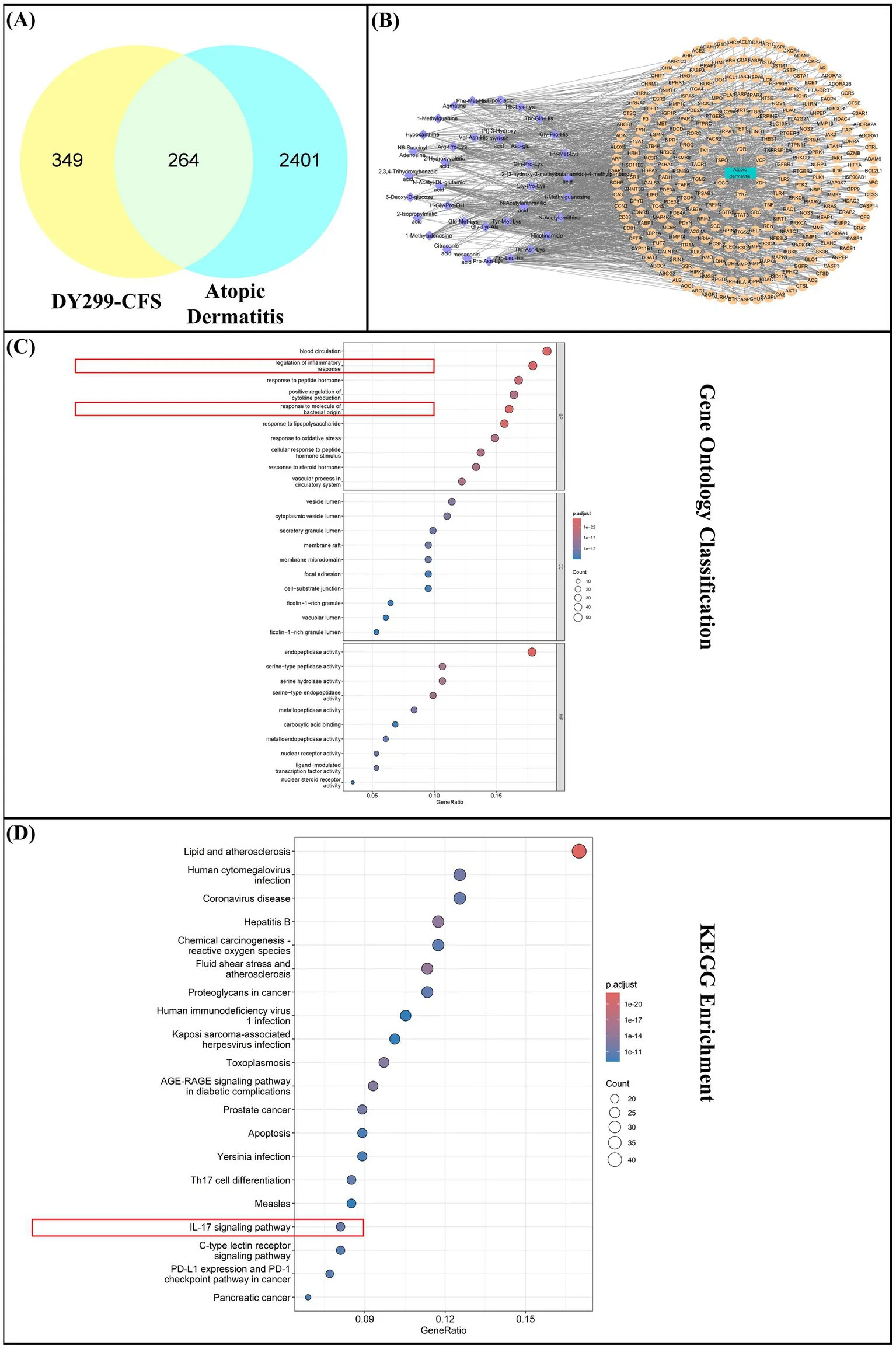
A comprehensive investigation of the network pharmacology of Atopic Dermatitis with inflammation with secondary metabolites of DY299-CFSs. **(A)** The Venn diagram illustrates the shared target genes between drugs and diseases. The size represents the quantity of target genes. The blue circle represents the target genes associated with Atopic Dermatitis. The yellow circle represents the target genes of the 36 components of DY299-CFSs. The overlapping area represents the shared target genes. **(B)** Compound-active ingredient-disease-target gene. The blue represents compounds, the yellow represents the targets of DY299-CFS acting on diseases. **(C)** GO analysis (top 10): Biological process (BP), Cellular component (CC), and Molecular function (MF). **(D)** Performing KEGG analysis to identify the top 20 enriched pathways. The size of each node indicates the number of target genes that are enriched, while the color of the node ranges from blue to red, indicating the *p*-value from high to low. The length of the node corresponds to the number of target genes that are enriched, meanwhile the color of the node ranges from blue to red, indicating the *p*-value from high to low.

## 4 Discussion

*Halophilic Bacillus* species are a group of bacteria capable of thriving in high-salinity environments, such as seawater and salt lakes, and are characterized by their pronounced salt tolerance. Numerous studies have demonstrated that metabolites produced by these *Halophilic Bacillus* strains possess anti-inflammatory, antibacterial, and immunomodulatory properties. In this study, a halotolerant strain designated *Bacillus halotolerans* DY299, which exhibits anti-biofilm activity against MRSA, was isolated from a water sample collected from the Arshan mud in Hulunbuir, Inner Mongolia. Based on 16 s rRNA gene sequence analysis, strain *Bacillus halotolerans* DY299 was identified as a member of the genus *Bacillus* and named *Bacillus halotolerans* DY299, showing the closest phylogenetic affiliation to *Bacillus halotolerans*. The species assignment was further corroborated by whole-genome ANI analysis, which showed 97.73% identity with *Bacillus halotolerans* NRRL B-41618, confidently confirming the strain as *Bacillus halotolerans*. Characterization of its growth properties revealed that strain *Bacillus halotolerans* DY299 displays broad thermal adaptability, being capable of growth at temperatures ranging from 10 to 45 °C. High-salt tolerance tests further demonstrated that *Bacillus halotolerans* DY299 can grow normally in the presence of 4–6% NaCl, confirming its pronounced halotolerance.

MRSA is one of the most common multi drug-resistant pathogens causing various infections (Abebe and Birhanu, 2023). The resistance of MRSA, coupled with the formation of biofilms, enables the bacteria to effectively evade the host immune system and antibiotics, thereby enhancing their resistance to antimicrobial agents and increasing the complexity of treatment (Kaushik et al., 2024). This study provides evidence for the inhibitory effects of DY299-CFS on both planktonic and biofilm-forming MRSA *in vitro* and *in vivo.*

Initially, the antibacterial activity of strain DY299-CFS against MRSA was evaluated. It was found that the DY299-CFS could inhibit the growth of MRSA. Crystal violet staining and scanning electron microscopy revealed that DY299-CFS inhibited the formation of MRSA biofilms and disrupted the integrity of the MRSA cell membrane in a concentration-dependent manner. Moreover, the number of viable bacteria within MRSA biofilms was significantly reduced after treatment with DY299-CFS. Numerous studies have reported that *Halophilic Bacillus* species exert antibacterial effects through direct killing of pathogens, disruption of biofilms, and interference with quorum sensing (QS) systems (Qiu et al., 2022; Murphy et al., 2024). For instance, highly *Halotolerant Bacillus subtilis* strain DZ17 and *Bacillus thuringiensis* strain DZ16, isolated from Algeria, were shown to interfere with the AI-2 and AHL QS systems, thereby regulating biofilm formation in bacteria (El Aichar et al., 2021). Consistent with these reports, our study confirmed that the DY299-CFS possesses potent biofilm-inhibitory activity against *S. aureus in vitro*.

Further *in vivo* experiments demonstrated that DY299-CFS not only inhibited the growth of MRSA on mouse skin but also alleviated the clinical symptoms of AD, as evidenced by reduced dermatitis scores, restored skin moisture content, and attenuated inflammatory responses in skin tissue. We also examined the effects of DY299-CFS on the expression of cytokines related to Th1, Th2, and Th17 immune responses. Compared with the model group, DY299-CFS treatment significantly reduced serum levels of IgE and IL-13. IgE is a key serological marker in atopic dermatitis, and its overproduction is closely linked to elevated levels of Th2 cytokines such as IL-4 and IL-13 (Renert-Yuval et al., 2021). IL-13 directly induces B cells to produce IgE, promotes excessive keratinocyte proliferation, disrupts the synthesis of aquaporins and filaggrin, damages the skin barrier, and stimulates pruriceptive nerve fibers. Together, these effects promote transepidermal water loss and trigger the “itch-scratch-barrier damage” vicious cycle (Tsoi et al., 2019). IFN-γ, a representative Th1 cytokine, further impairs skin barrier function, recruits macrophages, and is markedly upregulated during the chronic phase of dermatitis (Ren et al., 2022). Th17 cells, an important CD4^+^ T-cell subset, play a critical role in autoimmune diseases and host defense through the secretion of IL-17 (Zhao et al., 2023). Our results showed that, relative to the model group, the levels of IL-17 and IFN-γ in the DY299-CFS group were significantly decreased (*p* < 0.05), indicating that DY299-CFS can effectively suppress both Th1 and Th17 immune responses.

To elucidate the molecular mechanisms underlying the therapeutic effect of DY299-CFS on AD, we performed RNA-seq transcriptomic profiling to systematically identify key pathways and genes involved in inflammatory regulation. KEGG pathway enrichment analysis revealed the involvement of multiple signaling cascades, including the IL-17, cAMP, and CXCL pathways. After screening differentially expressed genes, eight candidates related to inflammation and immunity were selected for validation. The results confirmed that *Ccl3*, *Ccl4*, *Cxcl2*, *Lamc2*, *Il17ra*, and *Traf6* were significantly upregulated in the AD model group, and their expression was markedly reduced following DY299-CFS treatment, indicating that DY299-CFS can downregulate transcriptional regulators and attenuate cellular inflammatory responses. In the IL-17 signaling pathway, pro-inflammatory cytokines bind to IL-17RA and IL-17RC to form a receptor complex, which mediates the signal transduction of inflammatory target genes (Huangfu et al., 2023). This complex recruits the E3 ubiquitin ligase ACT1, which in turn activates TRAF6, leading to the downstream activation of the NF-κB and MAPK pathways (Song and Qian, 2013). Our results showed that DY299-CFS treatment decreased the expression of Il17ra and Traf6, suggesting that the anti-AD mechanism of DY299-CFS may involve, at least in part, suppression of the IL-17 signaling pathway. Moreover, a network pharmacology analysis of the active constituents in DY299-CFS indicated that these compounds may also exert therapeutic effects on AD through the IL-17 signaling pathways. The *in vivo* findings and the network pharmacology analysis thus corroborate each other, together demonstrating that DY299-CFS alleviates the clinical manifestations of atopic dermatitis by coordinately modulating inflammatory responses via multiple targets and IL-17 pathways.

Nevertheless, several limitations of this study should be acknowledged. First, the sample size in our *in vivo* experiments (n = 6 per group) was relatively small, which may limit the statistical power to detect subtle differences and constrains the generalizability of our conclusions. Additionally, the absence of a vehicle/solvent-only control group in the *in vivo* experiment limits our ability to fully attribute the therapeutic effects specifically to DY299-CFS, as opposed to solvent- or procedure-related non-specific responses. Of note, our *in vitro* antibacterial assays confirmed that the solvent alone exerted no inhibitory activity, and network pharmacology analysis ruled out significant contributions from culture medium-derived constituents; nonetheless, an *in vivo* vehicle control would have provided a more rigorous benchmark for evaluating the net pharmacological effect. Although the observed effects were statistically significant and consistent across multiple independent assays, future studies with larger cohorts are warranted to validate these results. Second, while we identified the IL-17 signaling pathways as key mediators through transcriptomic and network pharmacology analyses, the causal relationships between specific compounds in DY299-CFS and their direct molecular targets have not been experimentally verified. The bioactive constituents in the CFS are complex and may exert synergistic effects; therefore, bioactivity-guided fractionation and purification are necessary to isolate the individual active molecules responsible for the observed anti-biofilm and anti-inflammatory activities. Furthermore, the current study utilized the crude cell-free supernatant (DY299-CFS) rather than purified individual bioactive compounds. Although LC–MS/MS analysis identified 444 chemical components in DY299-CFS, the observed anti-biofilm and anti-inflammatory effects likely result from synergistic interactions among multiple constituents rather than the action of a single molecule. This complexity, while reflecting the natural pleiotropic nature of probiotic metabolites, also presents a challenge for precise mechanistic dissection and clinical standardization. We found that both heat-stable components and heat-labile molecules are involved in the inhibition of biofilm formation. Among the identified metabolites, we also detected numerous small-molecule short peptides (composed of 2–3 amino acids). However, it remains unclear whether these short peptides themselves exert the antibacterial and anti-inflammatory effects, or whether larger peptide molecules are responsible for the observed activities. The specific compound(s) responsible for the potent MRSA biofilm inhibition, as well as those mediating the suppression of IL-17 signaling pathway remain to be conclusively identified. Therefore, future work should prioritize bioactivity-guided fractionation to isolate the lead active molecules—potentially specific lipopeptides, bacteriocins, or other secondary metabolites—from DY299-CFS, followed by comprehensive structural elucidation and validation of their individual and combined therapeutic effects. Such efforts will be critical for translating the current proof-of-concept findings into a well-defined, chemically characterized therapeutic agent.

## 5 Conclusion

In this study, *Bacillus halotolerans* DY299 was isolated from Arshan water and identified via morphological and genomic analyses. DY299-CFS demonstrated potent anti-biofilm activity against MRSA *in vitro* and significantly alleviated AD-like skin lesions in MRSA-infected mice, as evidenced by improved dermatitis scores, skin hydration, reduced mast cell infiltration, and decreased serum levels of IgE, IL-13, IL-17, IFN-γ, and TNF-α. Mechanistically, transcriptomic and network pharmacology analyses revealed that the therapeutic effects are primarily mediated through coordinated modulation of the IL-17 signaling pathways. These findings establish *Bacillus halotolerans* DY299 as a promising candidate for biotherapeutic development against MRSA-associated skin infections.

However, this study has several limitations. The specific active compounds within DY299-CFS have not been purified or structurally characterized, and the direct molecular targets of these metabolites remain unvalidated, and the absence of a vehicle/solvent-only control group. Additionally, the study was conducted in a single murine model with a limited sample size, and long-term safety and efficacy have not been assessed.

Future research should focus on bioactivity-guided purification to identify the active molecules, validate their direct targets through molecular docking or affinity assays, and evaluate the translational potential in larger animal models and clinical settings. Further exploration of the strain’s genomic and metabolic capabilities may also expand its applicability to other biofilm-associated and inflammatory disorders.

## Data Availability

The datasets presented in this study can be found in online repositories. The names of the repository/repositories and accession number(s) can be found at: https://www.ncbi.nlm.nih.gov/, PRJNA1127323 and PRJNA224116.

## Footnotes

1
https://pubchem.ncbi.nlm.nih.gov/

2
http://www.swisstargetprediction.ch/

3
https://www.genecards.org/

4
https://www.ncbi.nlm.nih.gov/

5
https://ctdbase.org/

6
https://string-db.org/cgi/input.pl

7
https://jspecies.ribohost.com/jspeciesws/

## Glossary

MRSA: methicillin-resistant *Staphylococcus aureus*
LC–MS/MS: liquid chromatography–tandem mass spectrometry
CFS: cell-free supernatant
SCH: stratum corneum hydration
AD: atopic dermatitis
LB: luria bertani
RM: rich medium
PCR: polymerase chain reaction
NCBI: National Center for Biotechnology Information
UHPLC–MS/MS: ultra hight performance liquid chromatography–tandem mass spectrometry
PBS: phosphate buffered saline
CFU: colony forming unit
SEM: scanning electron microscope
SPF: specific pathogen-free
MUP: mupirocin
DNCB: 2,4-dinitrochlorobenzene
SCORAD: scoring atopic dermatitis
HE: hematoxylin and eosin
RNA-Seq: RNA sequencing
cDNA: complementary DNA
GO: gene ontology
KEGG: Kyoto Encyclopedia of Genes and Genomes
COG: clusters of orthologous genes
DEG: differentially expressed gene
CDS: coding sequence
GC: guanine-cytosine
POS: positive
NEG: negative
PLS-DA: partial least squares discriminant analysis
VIP: variable importance in the projection
FC: fold change
OMIM: databases online Mendelian inheritance in man databases
UHPLC-QE-MS: ultra-high performance liquid chromatography quadrupole-orbitrap mass spectrometry
TNF: tumor necrosis factor
IL-6: interleukin-6
IL-23: interleukin-23
JAKs: Janus kinases
ROR-γt: retinoid-related orphan receptor-γt
NF-κB: nuclear factor-κB
MAPK: mitogen-activated protein kinase
IL-17RA: interleukin-17 receptor A
TRAF: tumor necrosis factor receptor-associated factor
