# Supplementary material for: Anti-inflammatory effect of a Bacillus halotolerans DY299 strain isolated from Arshan on methicillin-resistant Staphylococcus aureus-induced atopic dermatitis in mouse
Source: Front Microbiol. 2026 Sep 22;17:1929286. doi: 10.3389/fmicb.2026.1929286 (PMC13638500; doi:10.3389/fmicb.2026.1929286)
Supplement: Supplementary file 1 [file Table_1.DOCX]

**Supplementary Table 1. The differential metabolites of DY299-CFS (log₂FC ≥ 1, VIP > 1, *p* < 0.05), their predicted targets from SwissTargetPrediction, and the number of overlapping targets with AD.**

| **Name** | **Number of predicted targets** | Predicted molecular targets | **Number of overlapping targets** | **AD-related overlapping targets** |
| --- | --- | --- | --- | --- |
| (R)-3-Hydroxy myristic acid | 81 | ZDHHC20, ZDHHC2, VDR, UGT2B7, TLR4, TERT, SHBG, SERPINA6, SCD, RORC, RELA, PTPRC, PTPN2, PTPN1, PTGFR, PTGES, PTGER2, PPARG, PPARD, PPARA, PLG, PHF8, PGR, NT5E, NR3C2, NR3C1, NR1H4, NPC1L1, MMP2, MAP3K5, LTB4R, LTA4H, KDM5C, KDM2A, IDO1, HSD17B3, HSD11B2, HSD11B1, HMGCR, HDAC3, HAO1, GSTK1, GRM5, GPR84, GPBAR1, G6PD, FOLH1, FFAR1, FABP5, FABP4, FABP3, FABP2, FABP1, FAAH, F3, F13A1, F10, ESR2, ESR1, ENPP2, EGLN1, CYP4F2, CYP4A11, CYP19A1, CISD1, CES2, CES1, CDC45, CDC25B, CDC25A, CAMK2A, CACNA2D2, CACNA2D1, CA2, CA1, AR, APLNR, AKR1B10, AKR1B1, ACLY, ACE | 36 | ACE, ACLY, AKR1B10, AR, CA2, ENPP2, ESR1, ESR2, F13A1, F3, FABP1, FABP3, FABP4, FABP5, HAO1, HMGCR, HSD11B1, HSD11B2, IDO1, LTA4H, LTB4R, MMP2, NR3C1, NR3C2, NT5E, PPARA, PPARD, PPARG, PTGER2, PTPRC, RELA, RORC, SCD, SERPINA6, TLR4, VDR |
| 2-Hydroxyvaleric acid | 12 | UGT2B7, CACNA2D2, PTPN2, FOLH1, CDC25B, EGLN1, APLNR, CA2, GRIK1, KDM2A, KDM5C, TET1 |  |  |
| 1-Methyladenosine | 53 | TYMP, TMIGD3, TMEM97, STING1, SRM, SRC, SLC6A3, SLC6A2, SLC29A1, SIGMAR1, SETD7, RAPGEF3, PRMT5, PRMT1, PNP, PLCG1, PDCD4, NT5E, NSD2, NNMT, MCL1, MAPK14, MAP3K7, INMT, HTR2C, HSPA8, HSPA5, HSP90B1, HRH1, GSK3A, FBP1, EZH2, EHMT2, EHMT1, EGFR, DPP4, DOT1L, CSNK2A1, CDA, CARM1, CA9, CA5A, CA4, CA2, CA1, AMD1, AHCY, ADORA3, ADORA2B, ADORA2A, ADORA1, ADK, ADA | 22 | ADA, ADORA1, ADORA2A, ADORA2B, ADORA3, AHCY, CA2, DPP4, EGFR, EHMT1, HRH1, HSP90B1, HSPA5, HSPA8, MAP3K7, MAPK14, MCL1, NT5E, PDCD4, SLC29A1, SRC, STING1 |
| 1-Methylguanine | 40 | XDH, VCP, TRPA1, TK1, SIRT3, PNP, PDE5A, PDE4A, PDE3B, PDE3A, PDE2A, PCSK9, OPRM1, OPRK1, NR2E1, MPO, MAPK10, MAOB, MAOA, LGMN, KDM1A, HTR1E, HPRT1, HDAC6, GDA, DYRK1A, CHIT1, CHIA, BRDT, BRD4, BRD3, BRD2, ADRA1D, ADRA1B, ADRA1A, ADORA3, ADORA2B, ADORA2A, ADORA1, ACHE | 19 | ADORA1, ADORA2A, ADORA2B, ADORA3, CHIA, CHIT1, LGMN, MPO, OPRK1, OPRM1, PCSK9, PDE2A, PDE3A, PDE3B, PDE4A, TK1, TRPA1, VCP, XDH |
| 1-Methylguanosine | 68 | VCP, TYMP, TMIGD3, TMEM97, TK1, TERT, STING1, SRC, SLC6A3, SLC6A2, SLC29A1, SLC28A2, SIGMAR1, SETD7, RPS6KA3, RAC1, RAB7A, PYGM, PRMT5, PRMT1, POLG, POLA1, PNP, PDE2A, PDCD4, NT5E, NSD2, NNMT, MDM2, MCL1, MAPK14, MAP3K7, KRAS, IMPDH2, HSPA8, HSPA5, HSP90B1, HRH1, HPRT1, HINT1, GSK3A, FPR2, FBP1, EZH2, EIF4E, EHMT2, EHMT1, EGFR, DPP4, DOT1L, DNMT1, DCPS, CDC42, CDA, CD38, CA9, CA4, CA2, CA1, BCHE, AMD1, AHCY, ADORA3, ADORA2B, ADORA2A, ADORA1, ADK, ADA | 31 | ADA, ADORA1, ADORA2A, ADORA2B, ADORA3, AHCY, BCHE, CA2, CD38, DNMT1, DPP4, EGFR, EHMT1, HRH1, HSP90B1, HSPA5, HSPA8, KRAS, MAP3K7, MAPK14, MCL1, NT5E, PDCD4, PDE2A, RAB7A, RAC1, SLC29A1, SRC, STING1, TK1, VCP |
| 2-(2-hydroxy-3-methylbutanamido)-4-methylpentanoic acid | 72 | XPNPEP2, XPNPEP1, XIAP, TGFBR1, SRD5A2, SLC6A5, SLC6A3, SLC5A1, SLC10A1, SIRT6, SIRT2, SIRT1, RNPEP, REN, PTGS2, PTGER4, PTGER2, PSMB5, PSMB2, PRKCA, PLA2G5, PLA2G4A, PLA2G2A, PLA2G10, PIK3CD, PEPD, NTSR2, NTSR1, NR1H4, MMP9, MMP3, MMP2, MMP1, MME, METAP2, LTA4H, LAP3, KEAP1, KDM6B, KDM3A, HSD17B3, HSD11B1, HLA-A, HDAC1, GLO1, FOLH1, FKBP1A, F2, EPHX2, ENPP2, ELANE, EGLN1, ECE1, DRD3, DPP4, DNPEP, CTSL, CTSD, CTSB, CSF1R, CNR2, CASP1, CAPN1, CA9, CA2, CA12, CA1, BIRC2, BACE1, AR, ACE2, ACE | 36 | ACE, ACE2, AR, BACE1, CA2, CASP1, CTSD, CTSL, DPP4, ECE1, ELANE, ENPP2, EPHX2, FKBP1A, GLO1, HDAC1, HLA-A, HSD11B1, KEAP1, LTA4H, MME, MMP1, MMP2, MMP3, MMP9, PIK3CD, PLA2G2A, PLA2G4A, PRKCA, PTGER2, PTGER4, PTGS2, REN, SIRT1, SLC10A1, TGFBR1 |
| 2, 3, 4-Trihydroxybenzoic acid | 37 | TYR, TTR, SRC, SNCA, SERPINE1, PTPN11, PTK2, NAPRT, MET, MAOB, MAOA, LDHB, LDHA, KDR, IGF1R, HMGB1, GRIN2B, GRIN2A, GPR35, GALNT2, FUT7, DPP4, CA9, CA7, CA6, CA5B, CA5A, CA4, CA3, CA2, CA14, CA12, CA1, BCL2L1, AURKB, ALK, ALB | 16 | ALB, BCL2L1, CA2, CA3, DPP4, FUT7, GALNT2, HMGB1, IGF1R, LDHA, LDHB, MET, PTK2, PTPN11, SERPINE1, SRC |
| 2-Hydroxyvaleric acid | 11 | PPARA, PPARG, CDC25A, PHF8, HAO1, FABP5, FABP3, TET3, HDAC3, FABP2, PTPN1 | 35 | ACE, ACLY, AKR1B10, AKR1C2, AKR1C3, AR, ASPH, CA2, CD81, ENPP2, FABP3, FABP4, FABP5, GRIN1, HAO1, HMGCR, HSD11B1, HSD11B2, IDO1, LTA4H, MME, MMP2, NR3C1, NR3C2, PPARA, PPARD, PPARG, PTGDR2, PTGER2, PTGS1, PTGS2, PTPRC, TET1, TGFBR1, VDR |
| 2-Isopropylmalic acid | 34 | TET3, TET2, TET1, SLC1A3, SLC1A2, SLC1A1, SLC13A5, PTPN2, PTPN1, PTGES, PTGER2, PHF8, KDM5C, KDM2A, IDO1, HSD11B1, HMGCR, HAO1, GRIK1, GPR84, FUCA1, FOLH1, FABP4, EGLN1, CDC25B, CDC25A, CAMK2A, CA9, CA2, CA12, CA1, APLNR, AKR1B1, ACLY | 9 | ACLY, CA2, FABP4, HAO1, HMGCR, HSD11B1, IDO1, PTGER2, TET1 |
| 2-Hydroxyvaleric acid | 36 | HSD11B1, HMGCR, CA12, PPARD, ACLY, CYP19A1, RNPEP, LTA4H, FFAR1, AKR1B10, GPR84, CISD1, PTGER2, PLG, NR1H4, IDO1, AR, ENPP2, AKR1B1, CPB2, AKR1C3, CYP4A11, VDR, HCAR2, DAO, CYP4F2, PTGES, TET2, NR3C2, PGR, SRD5A2, GPR17, FBP1, NR3C1, GABRR1, ACE |  |  |
| 6-Deoxy-D-glucose | 25 | TLR4, TLR2, SYNJ2, STAT3, PTPN1, PTGS2, PTGS1, PTAFR, NLRP3, MGAM, MAOB, LGALS8, LGALS4, LGALS3, HSP90AA1, GBA1, FUCA1, EPHX2, EIF4A1, CA9, CA2, CA12, CA1, ASGR1, AKR1B1 | 13 | ASGR1, CA2, EPHX2, GBA1, HSP90AA1, LGALS3, NLRP3, PTAFR, PTGS1, PTGS2, STAT3, TLR2, TLR4 |
| 2-Hydroxyvaleric acid | 32 | CA9, PTGDR2, PTGS2, CA4, KDM4E, KDM4C, F10, PTPRC, PTGS1, KDM8, CA1, ASPH, KDM5A, GPBAR1, AKR1C2, SLC6A12, MMP2, HSD11B2, FUCA1, GRIN1, GSTK1, CAMK2A, CPA1, KDM5B, TGFBR1, GRM5, MME, PAM, CACNA2D1, CD81, GRIK2, FABP4 |  |  |
| Agmatine | 44 | TYK2, SIGMAR1, RRM2, PKM, NOS3, NOS2, NOS1, LOXL2, JAK3, JAK2, JAK1, INMT, HTR3A, HRH4, HRH3, GABRR1, F2, DRD3, DPP9, DPP8, DPP7, DPP4, DHPS, DDAH1, CXCR4, CPN1, CPB2, CHRNA7, CHRM4, CHRM2, CHRM1, CASP2, CA9, CA7, CA6, CA5B, CA5A, CA4, CA3, CA2, CA14, CA12, CA1, AOC1 | 20 | AOC1, CA2, CA3, CHRM1, CHRM2, CHRNA7, CXCR4, DDAH1, DPP4, DPP9, HRH3, HRH4, JAK1, JAK2, JAK3, NOS1, NOS2, NOS3, RRM2, TYK2 |
| Arg-Pro-Lys | 72 | XPNPEP2, XPNPEP1, XIAP, WDR5, TPSAB1, TNFRSF10A, SLC5A1, SIRT6, SIRT5, SIRT2, SIRT1, REN, PTGS2, PRSS1, PRKCE, PREP, PLG, PLAU, PGA5, PEPD, PCSK6, NTSR2, NTSR1, NSD2, NRP1, MMP9, MMP8, MMP3, MMP2, MMP1, MME, MLLT3, MLLT1, MEP1B, MEN1, MASP2, LAP3, KLK1, KEAP1, HSD11B1, HLA-A, HGFAC, HDAC8, HDAC6, HDAC4, HDAC2, HDAC1, GLO1, FURIN, FOLH1, F2, F10, DPP8, DPP7, DPP4, DLG4, CTSL, CTSK, CTSE, CTSD, CTSB, CPB2, CPB1, CFB, CAPN1, BIRC2, BACE1, ANPEP, ADAM9, ADAM8, ADAM17, ACE | 33 | ACE, ADAM17, ADAM8, ADAM9, ANPEP, BACE1, CFB, CTSD, CTSE, CTSL, DPP4, GLO1, HDAC1, HDAC2, HDAC4, HLA-A, HSD11B1, KEAP1, MME, MMP1, MMP2, MMP3, MMP8, MMP9, NRP1, PLAU, PRKCE, PTGS2, REN, SIRT1, SIRT5, TNFRSF10A, TPSAB1 |
| Asp-glu | 40 | XIAP, SLC5A1, SLC1A2, SIRT1, REN, PTGS2, NTSR2, NFE2L2, MMP2, MMP1, MME, KEAP1, HLA-A, GZMB, GNPAT, GLO1, FOLH1, ENPP2, ENPEP, ELANE, DRD3, DPP4, DLG4, CTSL, CTSD, CTSB, CASP9, CASP8, CASP7, CASP6, CASP3, CASP2, CASP14, CASP10, CASP1, CAPN1, BACE1, APC, ADAM9, ACE | 25 | ACE, ADAM9, APC, BACE1, CASP1, CASP14, CASP3, CASP8, CASP9, CTSD, CTSL, DPP4, ELANE, ENPP2, GLO1, GZMB, HLA-A, KEAP1, MME, MMP1, MMP2, NFE2L2, PTGS2, REN, SIRT1 |
| cis-Aconitic acid | 2 | CA9, CA1 |  |  |
| Citraconic acid | 32 | TET3, TET2, TET1, PHF8, P4HTM, P4HA1, NFE2L2, LCK, KDM8, KDM6B, KDM5C, KDM5B, KDM5A, KDM4E, KDM4D, KDM4C, KDM4A, KDM3A, KDM2A, HIF1AN, HCAR2, FYN, FTO, FOLH1, EGLN3, EGLN2, EGLN1, CA9, CA1, ASPH, ALKBH5, AGER | 6 | ASPH, FYN, LCK, NFE2L2, P4HA1, TET1 |
| Gln-Pro-Lys | 91 | XPNPEP2, XPNPEP1, XIAP, WDR5, USP19, UBE2I, TNFRSF10A, THBS1, SLC6A3, SLC5A1, SIRT6, SIRT3, SIRT2, SIRT1, REN, PTGS2, PSMB5, PRSS1, PRKCE, PRKCD, PREP, PGA5, PEPD, PDF, P2RX7, NTSR2, NTSR1, NSD2, NRP1, NMT1, NFE2L2, MMP9, MMP3, MMP2, MMP1, MME, MLLT3, MLLT1, MAPK8, LAP3, KLKB1, KEAP1, HSD11B1, HLA-DRB3, HLA-A, HGFAC, HDAC6, HDAC4, HDAC3, HDAC2, HDAC1, GZMB, GNPAT, GLO1, FURIN, FOLH1, FAP, F2, F10, EP300, ENPP2, ENPEP, ELANE, DRD2, DPP9, DPP8, DPP7, DPP4, DLG4, CTSS, CTSL, CTSK, CTSE, CTSD, CTSB, CPB2, CPB1, CFB, CASP9, CASP8, CASP14, CAPN1, CACNA1H, BIRC3, BIRC2, BACE1, APC, ANPEP, ADAM9, ADAM8, ACE | 43 | ACE, ADAM8, ADAM9, ANPEP, APC, BACE1, CASP14, CASP8, CASP9, CFB, CTSD, CTSE, CTSL, CTSS, DPP4, DPP9, ELANE, ENPP2, FAP, GLO1, GZMB, HDAC1, HDAC2, HDAC4, HLA-A, HSD11B1, KEAP1, KLKB1, MAPK8, MME, MMP1, MMP2, MMP3, MMP9, NFE2L2, NRP1, PRKCD, PRKCE, PTGS2, REN, SIRT1, THBS1, TNFRSF10A |
| Glu-Met-Lys | 41 | ACE, XPNPEP2, XIAP, TACR3, SLC5A1, SIRT3, SIRT2, SIRT1, REN, PTPN1, PRKCE, PREP, NTSR2, NTSR1, MMP3, MMP1, MME, LAP3, KEAP1, KDM1A, HLA-DRB3, HLA-A, HGFAC, HDAC1, GSTP1, GSTM1, GSTK1, GSTA2, GSTA1, GLO1, FURIN, F2, ENPEP, DPP4, DLG4, CTSL, CTSD, CTSB, CASP14, CAPN1, BACE1 | 20 | ACE, BACE1, CASP14, CTSD, CTSL, DPP4, GLO1, GSTA1, GSTA2, GSTM1, GSTP1, HDAC1, HLA-A, KEAP1, MME, MMP1, MMP3, PRKCE, REN, SIRT1 |
| Gly-Pro-His | 52 | WDR5, TRHR, SLC6A5, SIRT2, SIRT1, REN, PTPN2, PTPN1, PPIA, PLK1, OXTR, NTSR2, NTSR1, NTMT1, MMP8, MMP3, MMP2, MMP13, MMP12, MMP10, MC4R, MC1R, LNPEP, IL1RN, IDE, HLA-DRB3, HLA-A, GRK2, F2, ERAP2, EPHX2, EDNRA, DPP7, CTSS, CTRL, CPB2, CPB1, CFB, CASP9, CASP5, CASP4, CASP14, CASP10, CAPN1, CA2, CA1, C3AR1, BRAF, BMP1, ANPEP, AGTR2, AGTR1 | 26 | ANPEP, BRAF, C3AR1, CA2, CASP14, CASP9, CFB, CTRL, CTSS, EDNRA, EPHX2, ERAP2, HLA-A, IL1RN, LNPEP, MC1R, MMP10, MMP12, MMP13, MMP2, MMP3, MMP8, PLK1, REN, SIRT1, ACE |
| Gly-Pro-Lys | 92 | XPNPEP2, XPNPEP1, XIAP, WDR5, USP19, TNFRSF10A, THBS1, SLC6A3, SLC5A1, SIRT6, SIRT3, SIRT2, SIRT1, REN, PTGS2, PSMB9, PSMB5, PRSS1, PRKCE, PRKCD, PREP, PLA2G5, PLA2G2A, PGA5, PEPD, PDF, NTSR2, NTSR1, NSD2, NRP1, NR1H4, NMT1, NFE2L2, MMP9, MMP8, MMP3, MMP2, MMP1, MME, MLLT3, MLLT1, MEP1B, LTA4H, LAP3, KLKB1, KEAP1, HSD11B1, HLA-DRB3, HLA-A, HGFAC, HDAC6, HDAC4, HDAC3, HDAC2, HDAC1, GZMB, GNPAT, GLO1, FURIN, FOLH1, FAP, F2, F10, ENPEP, ELANE, DRD3, DRD2, DPP9, DPP8, DPP7, DPP4, DLG4, CTSL, CTSE, CTSD, CTSB, CPB2, CPB1, CFB, CASP9, CASP8, CASP14, CASP1, CAPN1, CACNA1H, BIRC3, BIRC2, BACE1, APC, ANPEP, ADAM9, ACE | 44 | ACE, ADAM9, ANPEP, APC, BACE1, CASP1, CASP14, CASP8, CASP9, CFB, CTSD, CTSE, CTSL, DPP4, DPP9, ELANE, FAP, GLO1, GZMB, HDAC1, HDAC2, HDAC4, HLA-A, HSD11B1, KEAP1, KLKB1, LTA4H, MME, MMP1, MMP2, MMP3, MMP8, MMP9, NFE2L2, NRP1, PLA2G2A, PRKCD, PRKCE, PSMB9, PTGS2, REN, SIRT1, THBS1, TNFRSF10A |
| Gly-Tyr-Ala | 89 | YARS1, XIAP, TACR3, TACR2, TACR1, STAT3, SRC, SORT1, SLC15A1, SIRT1, REN, PTPN1, PTGS2, PIN1, PDYN, OPRM1, OPRK1, OPRD1, NTSR2, NTSR1, MMP9, MMP8, MMP3, MMP2, MMP14, MMP13, MMP12, MMP1, MME, MLNR, MLLT3, MLLT1, MDM4, MDM2, MAPK1, LNPEP, LCK, KLKB1, KLK1, KDM1A, ITGA4, IL1B, HSP90AB1, HSD11B1, HRH3, HMGCR, HLA-A, HIF1A, HDAC8, HDAC6, HDAC3, HDAC2, HDAC11, HDAC1, GRIA1, FYN, FPR1, F2, F11, F10, ERAP2, ERAP1, EPHX2, EED, EDNRA, CXCR4, CTSS, CTSL, CTSK, CTSD, CTSB, CPB2, CHRND, CCR5, CCKBR, CBX7, CBX4, CASP8, CASP1, CAPN1, ARRB1, APP, ANPEP, AGTR1, ADAM17, ACKR3, ACE, ABL1, ABCB1 | 51 | ABCB1, ACE, ACKR3, ADAM17, ANPEP, APP, CASP1, CASP8, CCR5, CTSD, CTSL, CTSS, CXCR4, EDNRA, EPHX2, ERAP1, ERAP2, FYN, HDAC1, HDAC2, HIF1A, HLA-A, HMGCR, HRH3, HSD11B1, HSP90AB1, IL1B, ITGA4, KLKB1, LCK, LNPEP, MAPK1, MME, MMP1, MMP12, MMP13, MMP14, MMP2, MMP3, MMP8, MMP9, OPRK1, OPRM1, PDYN, PTGS2, REN, SIRT1, SRC, STAT3, TACR1, TACR2 |
| H-Gly-Pro-OH | 91 | XPNPEP2, XPNPEP1, XIAP, TGFBR1, SLC6A3, SLC6A13, SLC6A12, SLC6A11, SLC6A1, SLC5A1, SLC1A2, SIRT6, SIRT2, SIRT1, RNPEP, REN, PTGS2, PRSS1, PRKCE, PREP, PLG, PLA2G5, PLA2G2A, PEPD, NTSR2, NTSR1, NRP1, NR1H4, NOS3, NOS2, NOS1, NFE2L2, MMP9, MMP8, MMP2, MMP1, MME, MLLT3, MLLT1, MEP1B, LTA4H, LAP3, KEAP1, HSD11B1, HLA-A, HGFAC, HDAC1, GSTK1, GRM3, GRIK2, GRIK1, GLO1, GBA1, GABRR1, FURIN, FAP, F2, F10, ENPP2, ENPEP, ELANE, DRD3, DRD2, DPP9, DPP8, DPP7, DPP4, DLG4, DDAH1, CTSL, CTSD, CTSB, CPN1, CPB2, CFB, CASP9, CASP8, CASP3, CASP14, CASP1, CAPN1, CA9, CA2, CA12, CA1, BIRC2, BACE1, APC, ANPEP, ACHE, ACE | 42 | ACE, ANPEP, APC, BACE1, CA2, CASP1, CASP14, CASP3, CASP8, CASP9, CFB, CTSD, CTSL, DDAH1, DPP4, DPP9, ELANE, ENPP2, FAP, GBA1, GLO1, HDAC1, HLA-A, HSD11B1, KEAP1, LTA4H, MME, MMP1, MMP2, MMP8, MMP9, NFE2L2, NOS1, NOS2, NOS3, NRP1, PLA2G2A, PRKCE, PTGS2, REN, SIRT1, TGFBR1 |
| His-Lys-Lys | 47 | WDR5, TRHR, SLC6A5, SIRT2, SIRT1, REN, PTPN2, PTPN1, PPIA, PLK1, OXTR, NTSR2, NTSR1, NTMT1, MMP8, MMP3, MMP2, MMP13, MMP12, MC4R, MC1R, LNPEP, IL1RN, IDE, HLA-DRB3, HLA-A, GRK2, F2, ERAP2, EPHX2, DPP7, CTSS, CTSL, CTRL, CPB2, CFB, CASP9, CASP5, CASP4, CASP14, CASP10, CAPN1, C3AR1, BRAF, ANPEP, AGTR2, AGTR1 | 23 | ANPEP, BRAF, C3AR1, CASP14, CASP9, CFB, CTRL, CTSL, CTSS, EPHX2, ERAP2, HLA-A, IL1RN, LNPEP, MC1R, MMP12, MMP13, MMP2, MMP3, MMP8, PLK1, REN, SIRT1 |
| Hypoxanthine | 36 | XDH, TYMP, TRPA1, TK1, SIRT3, PNP, PDE5A, PDE4A, PDE3B, PDE3A, PDE2A, NR2E1, LRRK2, LGMN, KDM5B, KDM4C, KDM4A, KDM1A, GSK3B, GDA, DYRK1A, DPYD, DAO, CYP19A1, CHIT1, CHIA, BRDT, BRD4, BRD3, BRD2, BACE1, ADORA3, ADORA2B, ADORA2A, ADORA1, ACHE | 17 | ADORA1, ADORA2A, ADORA2B, ADORA3, BACE1, CHIA, CHIT1, DPYD, GSK3B, LGMN, PDE2A, PDE3A, PDE3B, PDE4A, TK1, TRPA1, XDH |
| Lipoic acid | 17 | RNPEP, PTGS2, PTGER2, PPARG, PPARA, NR1H4, HSD11B1, GSTK1, GPR84, FOLH1, FABP5, FABP4, FABP3, CTSB, CPB2, AR, ACHE | 9 | AR, FABP3, FABP4, FABP5, HSD11B1, PPARA, PPARG, PTGER2, PTGS2 |
| mesaconic acid | 30 | TET3, TET2, TET1, PHF8, P4HTM, P4HA1, NFE2L2, KDM8, KDM6B, KDM5C, KDM5B, KDM5A, KDM4E, KDM4D, KDM4C, KDM4A, KDM3A, KDM2A, HIF1AN, HCAR2, FTO, FOLH1, EGLN3, EGLN2, EGLN1, CA9, CA1, ASPH, ALKBH5, AGER | 4 | ASPH, NFE2L2, P4HA1, TET1 |
| N6-Succinyl Adenosine | 47 | TSPO, TRDMT1, TMEM97, SMYD3, SMYD2, SLC6A3, SLC6A2, SLC29A1, SIGMAR1, SETD7, SETD2, RNMT, PRMT5, PRMT1, PPARG, PLCG1, NT5E, NSD2, NNMT, METTL3, MAP3K7, INMT, HTR7, HTR2C, HTR2B, EZH2, EHMT2, EHMT1, DPP4, DOT1L, DNMT3B, DNMT1, CTSS, CTSL, CTSK, CARM1, CA9, CA4, CA2, CA1, AMD1, AHCY, ADORA3, ADORA2B, ADORA2A, ADORA1, ADK | 17 | ADORA1, ADORA2A, ADORA2B, ADORA3, AHCY, CA2, CTSL, CTSS, DNMT1, DNMT3B, DPP4, EHMT1, MAP3K7, NT5E, PPARG, SLC29A1, TSPO |
| N-Acetylanthranilic acid | 95 | TTR, TRPM8, TRPM4, TRPM2, TRPA1, TERT, TAS2R14, SERPINE1, PTPRB, PTPN2, PTPN1, PTGS2, PTGS1, PTGES, PTGER4, PRSS1, PPARG, PPARD, PPARA, PLK3, PLK1, PLG, PLEC, PLAU, PLAT, PDE5A, PARP10, PARP1, OXTR, NR4A1, NR1H4, NOTUM, NAPRT, MPO, MMP2, MMP13, MCL1, MAPK8, MAPK10, MAOB, MAOA, LTC4S, LTB4R, LTA4H, LIPG, LIPC, LIG1, KMO, KLK1, KDM8, KDM6B, KDM5B, KDM5A, KDM4E, KDM4D, KDM4C, KDM4A, KCNK10, HSP90AA1, HSD11B1, HIPK2, HDAC8, HDAC6, HDAC1, HCAR2, GRIK1, FABP5, FABP4, F2, ESR2, ELANE, EGFR, DHODH, CNR2, CDC25C, CDC25B, CDC25A, CASP3, CA9, CA7, CA4, CA2, CA12, CA1, BRD4, BRAF, ASPH, APEX1, ALOX5, AKR1C3, AKR1C2, AKR1C1, AKR1B10, ABCG2, ABCC1 | 44 | ABCC1, ABCG2, AKR1B10, AKR1C2, AKR1C3, ALOX5, ASPH, BRAF, CA2, CASP3, EGFR, ELANE, ESR2, FABP4, FABP5, HDAC1, HIPK2, HSD11B1, HSP90AA1, KMO, LIPG, LTA4H, LTB4R, LTC4S, MAPK8, MCL1, MMP13, MMP2, MPO, NR4A1, PARP1, PLAT, PLAU, PLEC, PLK1, PPARA, PPARD, PPARG, PTGER4, PTGS1, PTGS2, SERPINE1, TRPA1, TRPM4 |
| N-Acetyl-DL-glutamic acid | 64 | UGCG, TGFBR1, TET2, SLC6A5, SLC6A13, SLC6A12, SLC6A11, SLC6A1, SLC1A2, RNPEP, REN, PTGS2, PTGER4, PTGER3, PTGER2, PLA2G5, PLA2G4A, PLA2G2A, PLA2G10, NR1H4, NAALAD2, MMP3, MMP2, MMP1, MME, LTA4H, LAP3, KEAP1, KDM4C, KDM4A, HSD11B1, HMGCR, HDAC1, GSR, GRM8, GRM6, GRM3, GRM2, GRIK5, GRIK3, GRIK2, GRIK1, GRIA4, GRIA1, GNPAT, GBA2, GBA1, GABRR1, FUCA1, FOLH1, F2, ENPP2, ECE1, DPP4, DLG4, CTSD, CASP3, CASP1, CAD, CA9, CA2, CA12, BACE1, ACE | 29 | ACE, BACE1, CA2, CASP1, CASP3, CTSD, DPP4, ECE1, ENPP2, GBA1, GSR, HDAC1, HMGCR, HSD11B1, KEAP1, LTA4H, MME, MMP1, MMP2, MMP3, PLA2G2A, PLA2G4A, PTGER2, PTGER3, PTGER4, PTGS2, REN, TGFBR1, UGCG |
| N-Acetylornithine | 92 | XIAP, TGFBR1, SLC6A5, SLC6A13, SLC6A12, SLC6A11, SLC6A1, SLC5A1, SLC1A2, SLC1A1, SLC10A1, SIRT2, RNPEP, REN, PTGS2, PTGER2, PRKCA, PLG, PLAU, PLA2G5, PLA2G2A, PIK3CD, PHF8, PEPD, NR3C1, NR1H4, NOS3, NOS2, NOS1, MMP9, MMP2, MMP1, MME, METAP2, MEP1B, LTA4H, LAP3, KEAP1, KDM5B, KDM4C, KDM4A, KDM2A, HSD17B3, HSD11B1, HDAC1, GSTK1, GRM8, GRM6, GRM3, GRM2, GRIK5, GRIK3, GRIK2, GRIK1, GRIA4, GRIA1, GNPAT, GLO1, GBA1, GABRR1, FOLH1, FAP, F2, EPHX2, ENPP2, ENPEP, ELANE, EGLN1, ECE1, DPP9, DPP8, DPP7, DPP4, DDAH1, CTSB, CPN1, CPB2, CPB1, CHRM1, CFB, CAPN1, CA9, CA2, CA12, CA1, BIRC2, BACE1, ARG1, ANPEP, ACHE, ACE2, ACE | 39 | ACE, ACE2, ANPEP, ARG1, BACE1, CA2, CFB, CHRM1, DDAH1, DPP4, DPP9, ECE1, ELANE, ENPP2, EPHX2, FAP, GBA1, GLO1, HDAC1, HSD11B1, KEAP1, LTA4H, MME, MMP1, MMP2, MMP9, NOS1, NOS2, NOS3, NR3C1, PIK3CD, PLA2G2A, PLAU, PRKCA, PTGER2, PTGS2, REN, SLC10A1, TGFBR1 |
| Nicotinamide | 92 | XDH, TYK2, TRPA1, TRIM33, TRIM24, TMEM97, TERT, TDO2, SRC, SIRT3, SIRT2, SIRT1, RPS6KB1, ROCK1, RIPK1, RAF1, PRKDC, PIM3, PIM2, PIM1, PIK3CD, PIK3CA, PARP1, P2RX7, NUDT1, NOS3, NOS2, NOS1, NNMT, NFATC1, MPO, MKNK2, MKNK1, MAPK14, MAP4K4, MAOA, KDR, JAK3, JAK2, JAK1, IMPDH2, IKBKB, IDO1, HTR7, HTR6, HTR2C, HTR1A, HSP90AA1, HPGDS, HDAC3, HDAC2, HDAC1, HCAR2, HASPIN, GSK3B, GPR84, EPHX2, EPHX1, EPHA2, EGFR, DYRK1A, DRD2, DPP4, DGAT1, CYP2A6, CYP19A1, CYP11B2, CYP11B1, CSNK2A1, CSF1R, CLK1, CHUK, CHRNA7, CHRM2, CHEK1, CFTR, CDK2, CDC7, CAPN1, CA9, CA2, CA1, BTK, BRAF, AURKB, AURKA, AHR, ADRA2A, ADRA1D, ADORA2A, ADORA1, ABL1 | 43 | ADORA1, ADORA2A, AHR, AURKA, BRAF, BTK, CA2, CFTR, CHRM2, CHRNA7, CHUK, CYP11B1, DGAT1, DPP4, EGFR, EPHX1, EPHX2, GSK3B, HDAC1, HDAC2, HPGDS, HSP90AA1, HTR1A, IDO1, IKBKB, JAK1, JAK2, JAK3, MAP4K4, MAPK14, MPO, NFATC1, NOS1, NOS2, NOS3, PARP1, PIK3CA, PIK3CD, SIRT1, SRC, TRPA1, TYK2, XDH |
| Phe-Met-His | 51 | TNF, TACR3, STAT3, SIRT3, SIRT2, SIRT1, SDCBP, REN, OXTR, OPRM1, OPRK1, OPRD1, NTMT1, NLN, MMP8, MMP3, MMP2, MMP13, MMP12, MMP10, MC4R, MC3R, MC1R, MAGI3, LNPEP, KLKB1, IL1RN, IDE, HRH1, HLA-A, GRPR, GHSR, FPR2, FPR1, FDFT1, F2, ERAP2, EPHX2, EDNRA, CTSS, CTSD, CTRL, CPB2, CCKBR, CA2, CA1, BACE1, ANPEP, AGTR2, AGTR1, ACKR3 | 30 | ACKR3, ANPEP, BACE1, CA2, CTRL, CTSD, CTSS, EDNRA, EPHX2, ERAP2, FDFT1, HLA-A, HRH1, IL1RN, KLKB1, LNPEP, MC1R, MC3R, MMP10, MMP12, MMP13, MMP2, MMP3, MMP8, OPRK1, OPRM1, REN, SIRT1, STAT3, TNF |
| Pro-Asn-Lys | 90 | XPNPEP2, XPNPEP1, XIAP, WDR5, UBE2I, TNFRSF10A, THBS1, TGM2, SPSB2, SLC5A1, SIRT6, SIRT5, SIRT3, SIRT2, SIRT1, REN, PTGS2, PRSS1, PRKCE, PREP, PLG, PGA5, PEPD, PADI4, PADI1, NTSR2, NTSR1, NRP1, NPR1, NFE2L2, MMP9, MMP8, MMP3, MMP2, MMP1, MME, MLLT3, MLLT1, MASP2, MAPK8, LAP3, KEAP1, HSD11B1, HLA-DRB3, HLA-DRB1, HLA-A, HGFAC, HDAC6, HDAC4, HDAC3, HDAC2, HDAC1, GZMB, GNPAT, GLO1, FURIN, FHIT, FAP, F2, F10, ENPEP, ELANE, DPP9, DPP4, DLG4, CTSS, CTSL, CTSK, CTSE, CTSD, CTSB, CFB, CASP9, CASP8, CASP7, CASP6, CASP5, CASP3, CASP2, CASP14, CASP10, CASP1, CAPN1, BIRC2, BACE1, APC, ANPEP, ADAM9, ADAM8, ACE | 48 | ACE, ADAM8, ADAM9, ANPEP, APC, BACE1, CASP1, CASP14, CASP3, CASP8, CASP9, CFB, CTSD, CTSE, CTSL, CTSS, DPP4, DPP9, ELANE, FAP, GLO1, GZMB, HDAC1, HDAC2, HDAC4, HLA-A, HLA-DRB1, HSD11B1, KEAP1, MAPK8, MME, MMP1, MMP2, MMP3, MMP8, MMP9, NFE2L2, NPR1, NRP1, PADI1, PRKCE, PTGS2, REN, SIRT1, SIRT5, TGM2, THBS1, TNFRSF10A |
| Pseudouridine | 1 | PNP |  |  |
| Thr-Asn-Lys | 89 | XPNPEP2, XPNPEP1, XIAP, WDR5, USP19, UBE2I, TNFRSF10A, THBS1, TGM2, SPSB2, SLC6A3, SLC5A1, SIRT6, SIRT5, SIRT3, SIRT2, SIRT1, REN, PTGS2, PSMB5, PRSS1, PRKCE, PRKCD, PREP, PLG, PGA5, PEPD, PADI4, PADI1, NTSR2, NTSR1, NRP1, NPR1, NMT1, NFE2L2, MMP9, MMP8, MMP3, MMP2, MMP1, MME, MLLT3, MLLT1, MAPK8, LAP3, KEAP1, HSD11B1, HLA-DRB3, HLA-DRB1, HLA-A, HGFAC, HDAC1, GZMB, GNPAT, GLO1, FURIN, FAP, F2, F12, F10, ENPEP, ELANE, DPP9, DPP4, DLG4, CTSS, CTSL, CTSK, CTSE, CTSD, CTSB, CASP9, CASP8, CASP7, CASP6, CASP5, CASP3, CASP2, CASP14, CASP10, CASP1, CAPN1, BIRC2, BACE1, APC, ANPEP, ADAM9, ADAM8, ACE | 46 | ACE, ADAM8, ADAM9, ANPEP, APC, BACE1, CASP1, CASP14, CASP3, CASP8, CASP9, CTSD, CTSE, CTSL, CTSS, DPP4, DPP9, ELANE, FAP, GLO1, GZMB, HDAC1, HLA-A, HLA-DRB1, HSD11B1, KEAP1, MAPK8, MME, MMP1, MMP2, MMP3, MMP8, MMP9, NFE2L2, NPR1, NRP1, PADI1, PRKCD, PRKCE, PTGS2, REN, SIRT1, SIRT5, TGM2, THBS1, TNFRSF10A |
| Thr-Gln-His | 46 | WDR5, TRHR, SLC6A5, SIRT2, SIRT1, REN, PTPN2, PTPN1, PPIA, PLK1, NTSR2, NTSR1, MMP8, MMP3, MMP2, MMP13, MMP12, MMP10, MC4R, MC1R, MAGI3, LNPEP, IL1RN, IDE, HLA-DRB3, HLA-A, GRK2, F2, ERAP2, EPHX2, EDNRA, CTSD, CTRL, CPB2, CPB1, CFB, CASP9, CASP5, CASP4, CASP14, CASP10, C3AR1, BRAF, ANPEP, AGTR2, AGTR1 | 24 | ANPEP, BRAF, C3AR1, CASP14, CASP9, CFB, CTRL, CTSD, EDNRA, EPHX2, ERAP2, HLA-A, IL1RN, LNPEP, MC1R, MMP10, MMP12, MMP13, MMP2, MMP3, MMP8, PLK1, REN, SIRT1 |
| Thr-Met-Lys | 56 | XPNPEP2, XIAP, TNFRSF10A, THBS1, TACR3, SLC5A1, SIRT6, SIRT3, SIRT2, SIRT1, REN, PTPN2, PTPN1, PTGS2, PSMB5, PRKCE, PREP, PLK1, PGA5, NTSR2, NTSR1, NMT1, MMP9, MMP3, MMP1, MME, MLLT1, LTA4H, LAP3, KEAP1, KDM1A, HLA-DRB3, HLA-A, HGFAC, HDAC8, HDAC1, GSTP1, GSTM1, GSTA2, GSTA1, FURIN, FPR1, F2, ENPEP, DPP4, DLG4, CTSL, CTSD, CTSC, CTSB, CAPN1, BIRC2, BACE1, ANPEP, ADAM9, ACE | 27 | ACE, ADAM9, ANPEP, BACE1, CTSC, CTSD, CTSL, DPP4, GSTA1, GSTA2, GSTM1, GSTP1, HDAC1, HLA-A, KEAP1, LTA4H, MME, MMP1, MMP3, MMP9, PLK1, PRKCE, PTGS2, REN, SIRT1, THBS1, TNFRSF10A |
| Trp-Leu-His | 92 | TNF, TACR3, TACR2, TACR1, ST3GAL1, SSTR5, SSTR4, SSTR3, SSTR2, SSTR1, SOAT1, SDCBP, REN, RBM17, PTGS2, PSMB8, PPIA, PPARG, PLG, PIN1, OXTR, OPRM1, OPRK1, OPRD1, NTSR1, MMP9, MMP8, MMP7, MMP3, MMP2, MMP13, MMP12, MMP1, MLLT3, MLLT1, MC5R, MC4R, MC3R, MC1R, MAGI3, LNPEP, KEAP1, IL1RN, IDE, HRH1, HLA-A, HDAC8, HDAC6, HDAC4, HDAC3, HDAC2, HDAC11, HDAC10, HDAC1, GRPR, GID4, GHSR, FPR2, F2, ERAP2, ERAP1, EPHX2, EPHB6, EPHB4, EPHA7, EPHA2, EDNRB, EDNRA, DPP3, CTSS, CTSL, CTSD, CTSB, CTRL, CCN2, CCKBR, CCKAR, CBX7, CASP9, CASP8, CASP7, CAPN1, CA2, CA1, C3AR1, BCHE, APC, ANPEP, AGTR2, AGTR1, ACKR3, ACE | 47 | ACE, ACKR3, ANPEP, APC, BCHE, C3AR1, CA2, CASP8, CASP9, CCN2, CTRL, CTSD, CTSL, CTSS, EDNRA, EDNRB, EPHX2, ERAP1, ERAP2, HDAC1, HDAC2, HDAC4, HLA-A, HRH1, IL1RN, KEAP1, LNPEP, MC1R, MC3R, MC5R, MMP1, MMP12, MMP13, MMP2, MMP3, MMP8, MMP9, OPRK1, OPRM1, PPARG, PSMB8, PTGS2, REN, SSTR5, TACR1, TACR2, TNF |
| Tyr-Met-Lys | 93 | YARS1, XIAP, UTS2R, TACR3, TACR2, TACR1, STAT3, SRC, SORT1, SIRT1, REN, PTGS2, PRSS1, PROC, PPP5C, PPP1CA, PLG, PIN1, PDYN, OXTR, OPRM1, OPRK1, OPRD1, NTSR2, NTSR1, NTMT1, NPFFR2, MMP9, MMP8, MMP3, MMP1, MME, MLNR, MLLT3, MLLT1, MAPK1, LNPEP, LCK, KLK7, ITGA4, IL1RN, HSPA2, HSP90AB1, HSD11B1, HMGCR, HLA-DRB3, HLA-DRB1, HLA-A, HIF1A, HDAC8, HDAC6, HDAC4, HDAC3, HDAC2, HDAC11, HDAC10, HDAC1, GRIA1, FPR2, FPR1, FDFT1, F2, F11, F10, ERAP2, ERAP1, EPHX2, EED, ECE1, CTSL, CTSD, CTSB, CPB2, CPA1, CHRND, CCKBR, CBX7, CASP8, CASP1, C5AR1, BACE1, AVPR2, AVPR1B, AVPR1A, ARRB1, APP, APC, ANPEP, AKT1, AGTR1, ACKR3, ACE, ABCB1 | 50 | ABCB1, ACE, ACKR3, AKT1, ANPEP, APC, APP, BACE1, C5AR1, CASP1, CASP8, CTSD, CTSL, ECE1, EPHX2, ERAP1, ERAP2, FDFT1, HDAC1, HDAC2, HDAC4, HIF1A, HLA-A, HLA-DRB1, HMGCR, HSD11B1, HSP90AB1, HSPA2, IL1RN, ITGA4, KLK7, LCK, LNPEP, MAPK1, MME, MMP1, MMP3, MMP8, MMP9, OPRK1, OPRM1, PDYN, PROC, PTGS2, REN, SIRT1, SRC, STAT3, TACR1, TACR2 |
| Val-Asn-His | 41 | WDR5, TRHR, SLC6A5, SIRT2, SIRT1, REN, PTPN2, PTPN1, PPIA, PLK1, OPRD1, NTSR2, NTSR1, MMP8, MMP3, MMP2, MMP13, MC4R, MC1R, LNPEP, IL1RN, IDE, HLA-DRB3, HLA-A, GRK2, ERAP2, EPHX2, EDNRA, CTRL, CPB2, CPB1, CFB, CASP9, CASP5, CASP4, CASP14, CASP10, CA2, CA1, C3AR1, BRAF, ANPEP, AGTR2, AGTR1 | 22 | ANPEP, BRAF, C3AR1, CA2, CASP14, CASP9, CFB, CTRL, EDNRA, EPHX2, ERAP2, HLA-A, IL1RN, LNPEP, MC1R, MMP13, MMP2, MMP3, MMP8, PLK1, REN, SIRT1 |
